# Pyrazole Scaffold Synthesis, Functionalization, and Applications in Alzheimer’s Disease and Parkinson’s Disease Treatment (2011–2020)

**DOI:** 10.3390/molecules26051202

**Published:** 2021-02-24

**Authors:** Xuefei Li, Yanbo Yu, Zhude Tu

**Affiliations:** Department of Radiology, Washington University School of Medicine, 510 S. Kingshighway Boulevard St. Louis, MO, 63110, USA; xuefeili@wustl.edu (X.L.); yanboyu@wustl.edu (Y.Y.)

**Keywords:** pyrazole, synthesis, functionalization, heterocyclic, neurodegeneration, Alzheimer’s disease, Parkinson’s disease, inhibitor, antagonist, biological activity

## Abstract

The remarkable prevalence of pyrazole scaffolds in a versatile array of bioactive molecules ranging from apixaban, an anticoagulant used to treat and prevent blood clots and stroke, to bixafen, a pyrazole-carboxamide fungicide used to control diseases of rapeseed and cereal plants, has encouraged both medicinal and organic chemists to explore new methods in developing pyrazole-containing compounds for different applications. Although numerous synthetic strategies have been developed in the last 10 years, there has not been a comprehensive overview of synthesis and the implication of recent advances for treating neurodegenerative disease. This review first presents the advances in pyrazole scaffold synthesis and their functionalization that have been published during the last decade (2011–2020). We then narrow the focus to the application of these strategies in the development of therapeutics for neurodegenerative diseases, particularly for Alzheimer’s disease (AD) and Parkinson’s disease (PD).

## 1. Introduction

**Figure 1:**
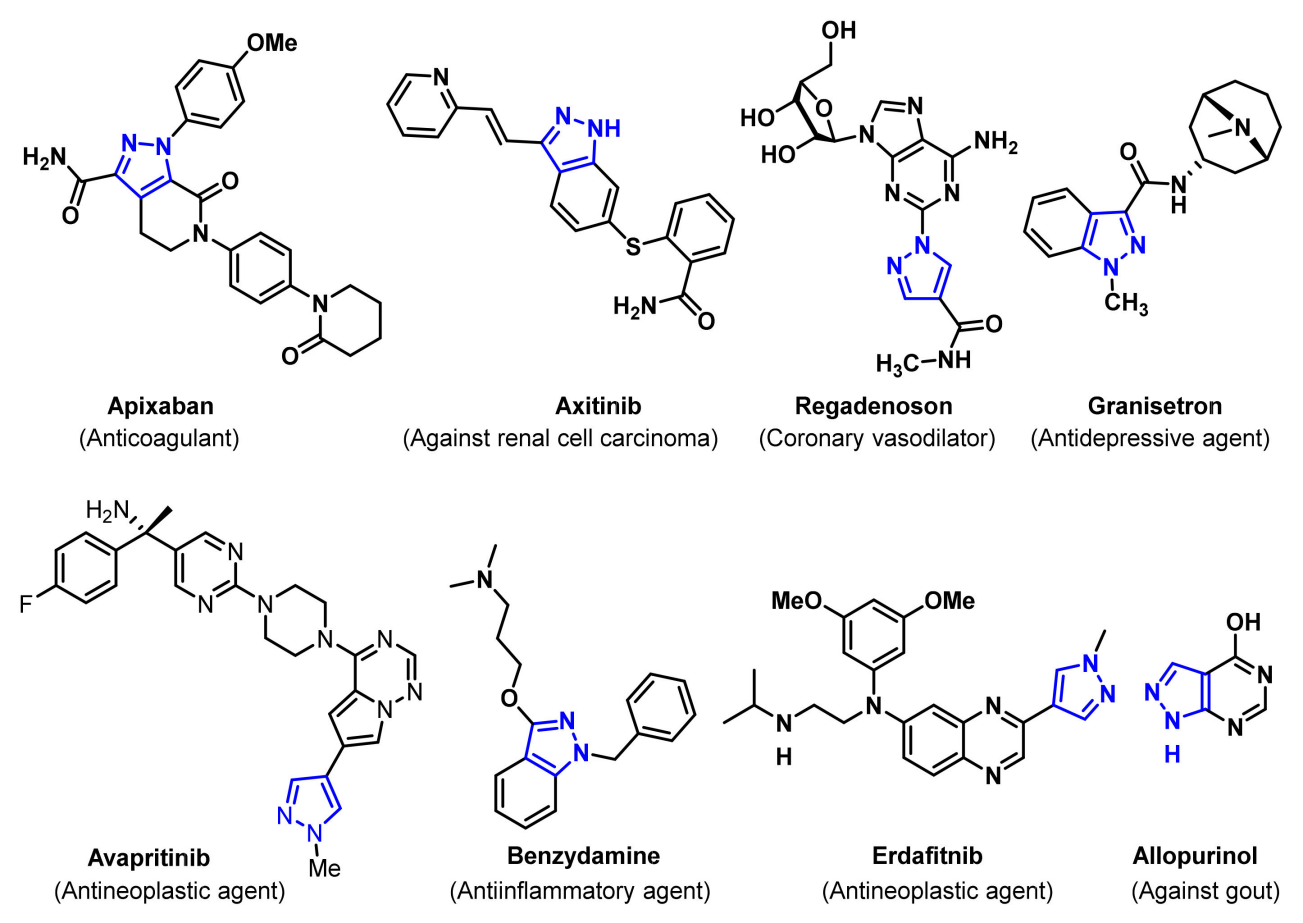
Representative FDA-approved pyrazole-containing medicines. FDA: U.S. Food and Drug Administration.

Pyrazole compounds contain a five-membered aromatic ring composed of three carbon atoms and two adjacent nitrogen atoms. The shifting C-N double bond inside the heterocycle endows pyrazoles with tautomerism. Consequently, regioselectivity can be a challenge in the synthesis and purification of *N*-substituted and asymmetric pyrazoles. Although a pyrazole core is rare in natural compounds, artificial pyrazole derivatives are prevalent in diverse fields. Applications range from agrochemicals [1] to therapeutics. At least thirty-three pyrazole-containing medicines have been marketed to alleviate or treat diseases ranging from bacterial infections to cancer and neurologic disorder (Figure 1) [2,3]. For example, the anticoagulant apixaban is used to prevent serious blood clots that may cause stroke, heart attack, and is also prescribed to patients with an abnormal heartbeat (atrial fibrillation) or hip/knee joint replacement surgery; in 2018, apixaban was the second most popular blockbuster drug [4]. Investigators in both academic institutions and pharmaceutical industries have put tremendous efforts into the exploration of new pyrazole scaffolds for drug development. This review, building upon previous literature, reviews [3,5] and presents progress in the chemical synthesis of pyrazole scaffold molecules and the functionalization of pyrazole derivatives from 2011 to 2020. We then focus on pyrazole-containing small biomolecules developed as therapeutic candidates for treating neurodegenerative diseases, particularly those that target pathologies found in Alzheimer’s disease (AD) and Parkinson’s disease (PD). Fluorine-18 radiotracers composed of pyrazole scaffolds have been recently reviewed by Gomes and his colleagues [6] and will not be discussed in this review.

## 2. The Synthesis of Pyrazole Scaffold Molecules

**Scheme 1:**
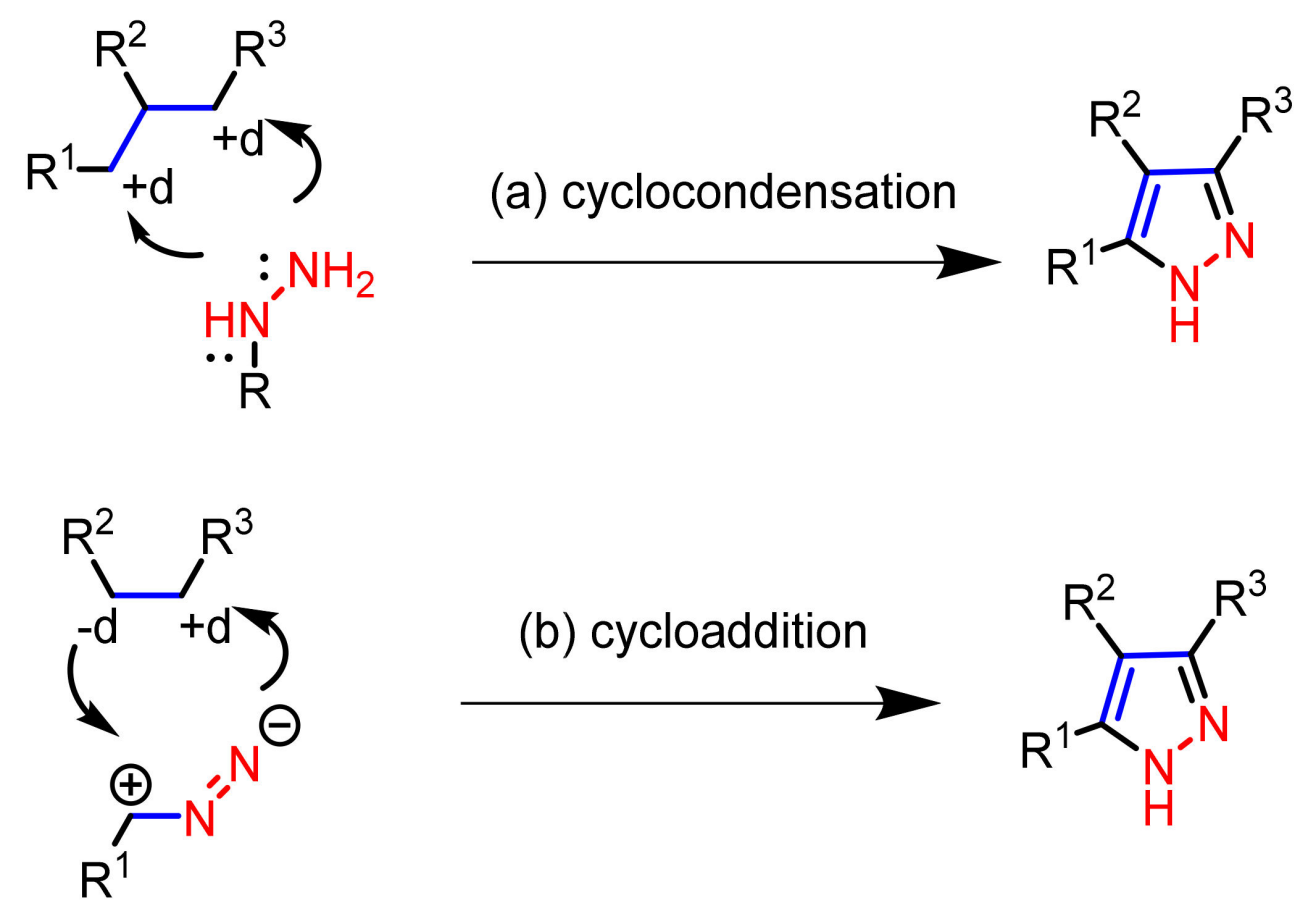
Conventional pyrazole synthetic strategies. (**a**): cyclocondensation; (**b**): cycloaddition.

**Scheme 2:**
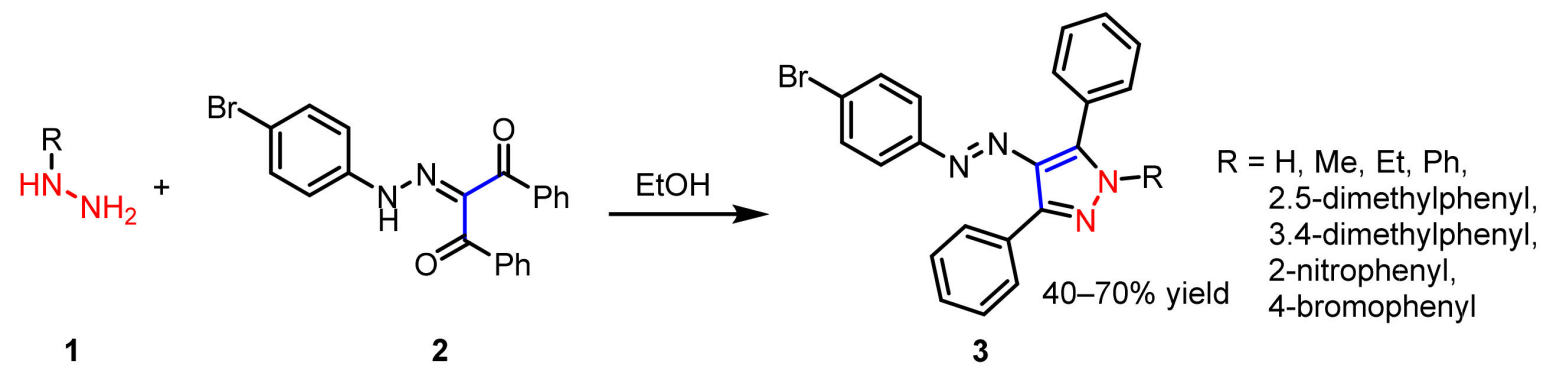
Bioactive molecules synthesis by cyclocondensation.

The synthesis of substituted pyrazoles has been accomplished by two strategies [3,5]: (1) the cyclocondensation of hydrazines with 1,3-dicarbonyl compounds or their synthetic 1,3-dielectrophilic equivalents (Scheme 1a), and (2) the cycloaddition of 1,3-dipoles to dipolarophiles (Scheme 1b). These two conventional strategies were recently enriched by additional approaches including multicomponent one-pot processes, photoredox reactions, and transition-metal catalyzed reactions. In this section, we will first discuss cyclocondensation, followed with cycloaddition. In addition, new approaches are also discussed.

### 2.1. Cyclocondensation of Hydrazines with 1,3-Dielectrophilic Derivatives

**Figure 2:**
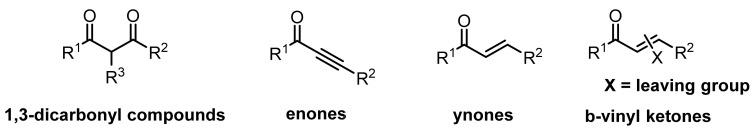
Structures of 1,3-dielectrophilic derivatives.

Substituted or nonsubstituted hydrazines are readily available as [*NN*] synthons for the synthesis of pyrazole derivatives. Pyrazole molecules can be generated through hydrazines reacting with 1,3-dielectrophilic units such as 1,3-dicarbonyl or with α,β-unsaturated carbonyl compounds; the latter structures include enones, ynones, and vinyl ketones bearing a leaving group (Figure 2) [3].

#### 2.1.1. Cyclocondensation of Hydrazines with 1,3-Dicarbonyl and Related Compounds

1,3-diketones, β-ketoesters, 2,4-diketoesters, and related synthetic equivalents can condense efficiently with hydrazines to generate substituted pyrazoles. Based on this strategy, a series of potent carbonic anhydrase, α-glycosidase, and cholinesterase enzymes inhibitors **1** were synthesized via the cyclocondensation of the 1,3-diketone and appropriate hydrazines (Scheme 2) [7].

**Scheme 3:**
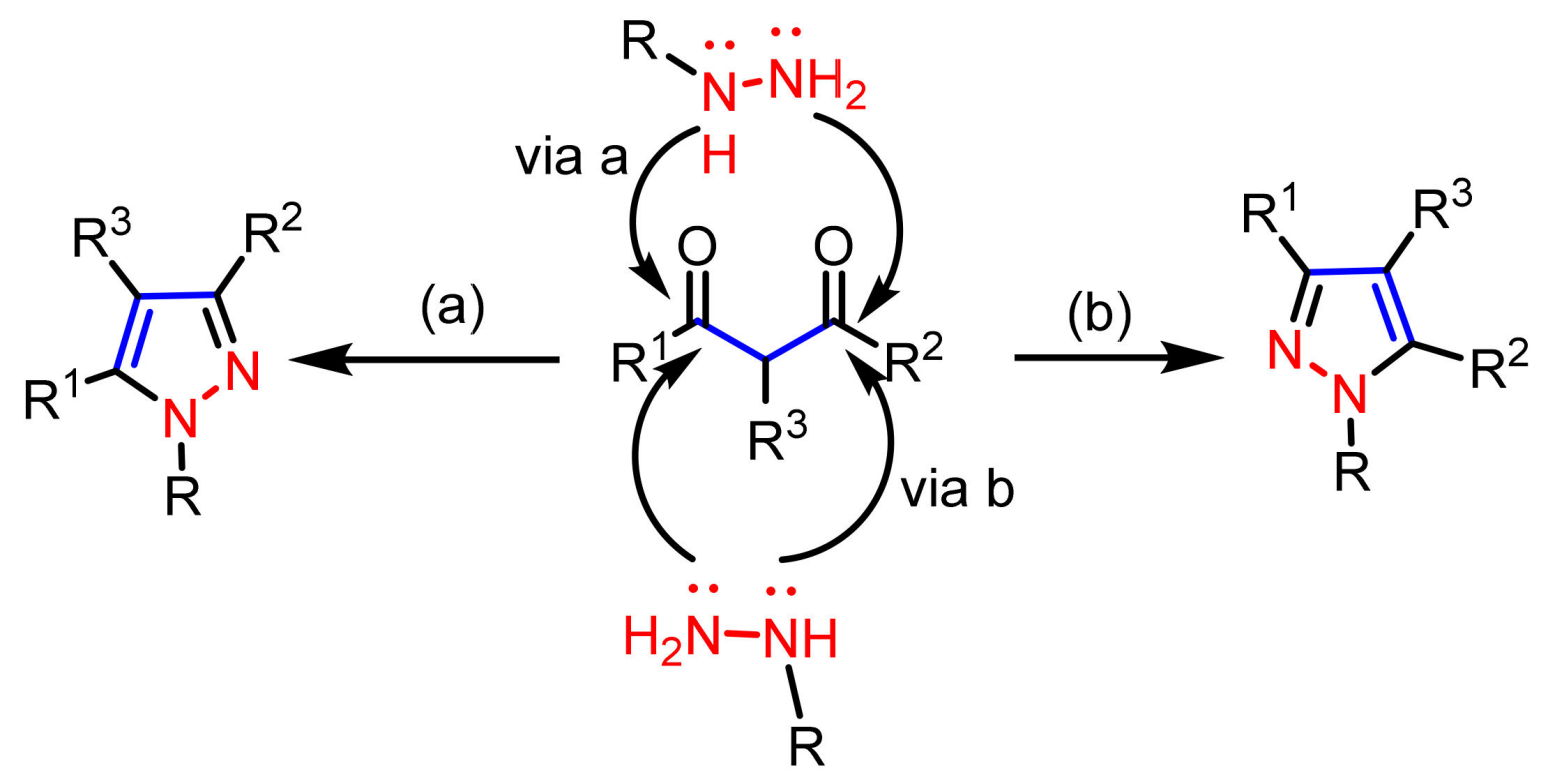
Regioselectivity of pyrazoles synthesis via cyclocondensation.

However, a mixture of two regioisomers is produced in most cases when unsymmetrical 1,3-dicarbonyl compounds (R^1^ ≠ R^2^), and substituted hydrazines are used for cyclocondensation (Scheme 3). Moreover, this kind of cyclocondensation is generally accomplished in a strongly acidic medium (i.e., fluoroboric acid [8]), thus the reaction solution is corrosive and not environmentally-friendly.

**Scheme 4:**
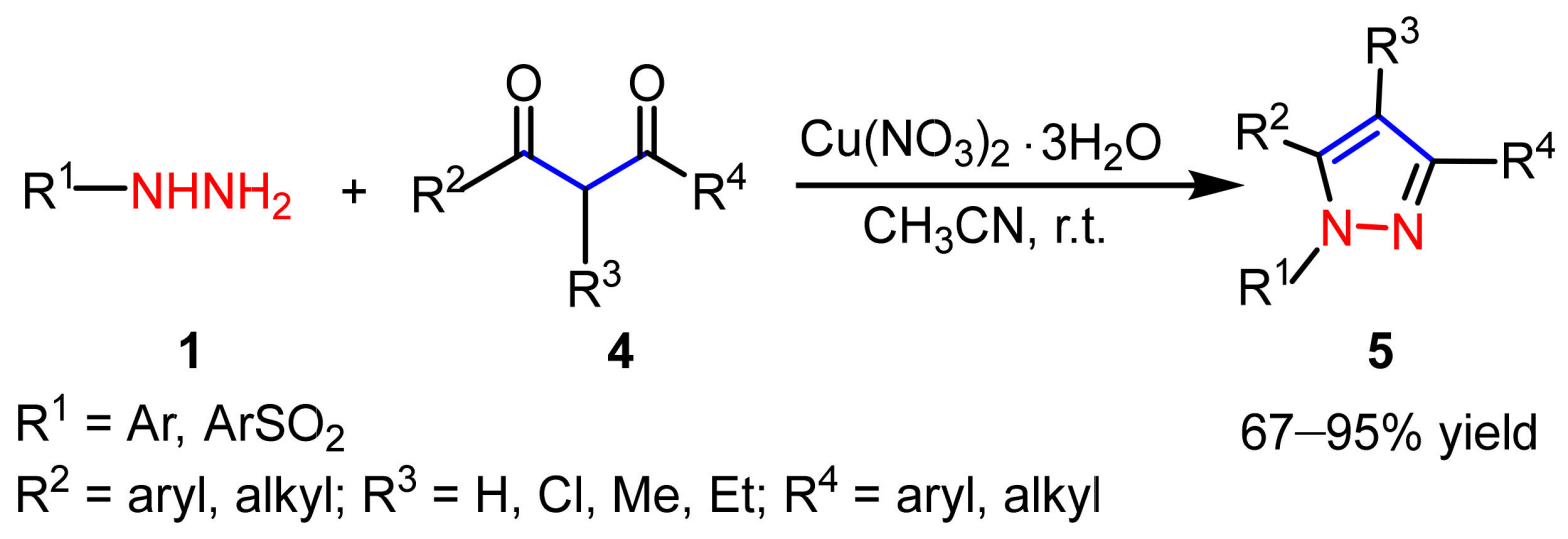
Cu-catalyzed cyclocondensation for pyrazole synthesis.

A mild and acid-free condensation of 1,3-diketones with substituted hydrazines to generate the 1,3,5-trisubstituted and fully substituted pyrazoles was reported by Wang and co-workers [9]. The optimal conditions were obtained when using copper (II) nitrate as the catalyst, providing the cyclocondensation products at room temperature in less than 60 min. More importantly, this method provided highly regioselective products with good yields (Scheme 4).

**Scheme 5:**
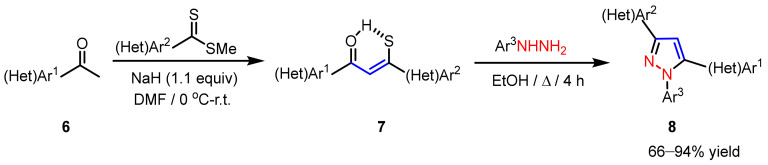
1,3-monothiodiketones as substrates for pyrazole synthesis.

As a novel derivative of 1,3-dicarbonyl compounds, 1,3-monothiodiketones are good complements for preparing unsymmetrically substituted 1-aryl-3,5-bis(het)arylpyrazoles with excellent regioselectivity and high yield (Scheme 5) [10].

**Scheme 6:**
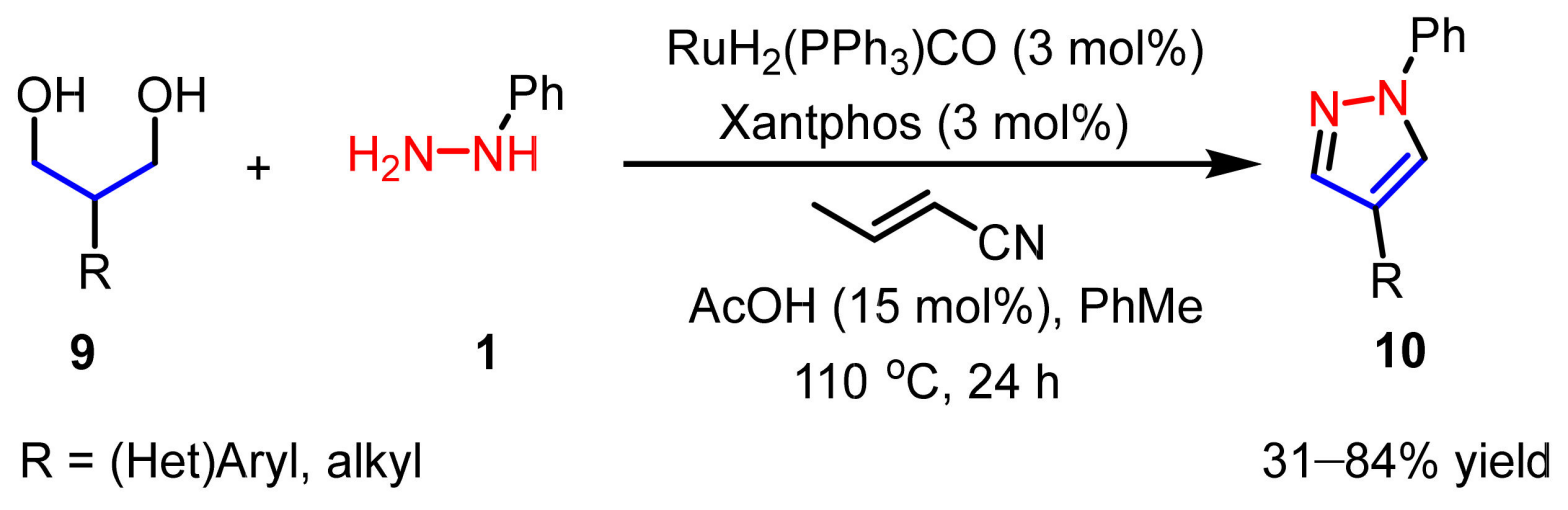
1,3-diols as substrates for pyrazole synthesis.

Despite the efficiency and flexibility of using 1,3-dicarbonyl compounds as substrates, the synthetic inconvenience and inherent instability of many 1,3-dicarbonyls, particularly dialdehydes, limits the synthesis of some substituted pyrazoles. To overcome this challenge, Schmitt et al. reported the facile generation of 1,4-disubstituted pyrazoles when using ruthenium as a catalyst with 1,3-diols to replace 1,3-dicarbonyl compounds [11]. In this reaction, crotonitrile was used as the reductant to accept hydrogen transfer from ruthenium dihydrides (Scheme 6).

#### 2.1.2. Cyclocondensation of Hydrazines with Enones and Related Compounds

**Scheme 7:**
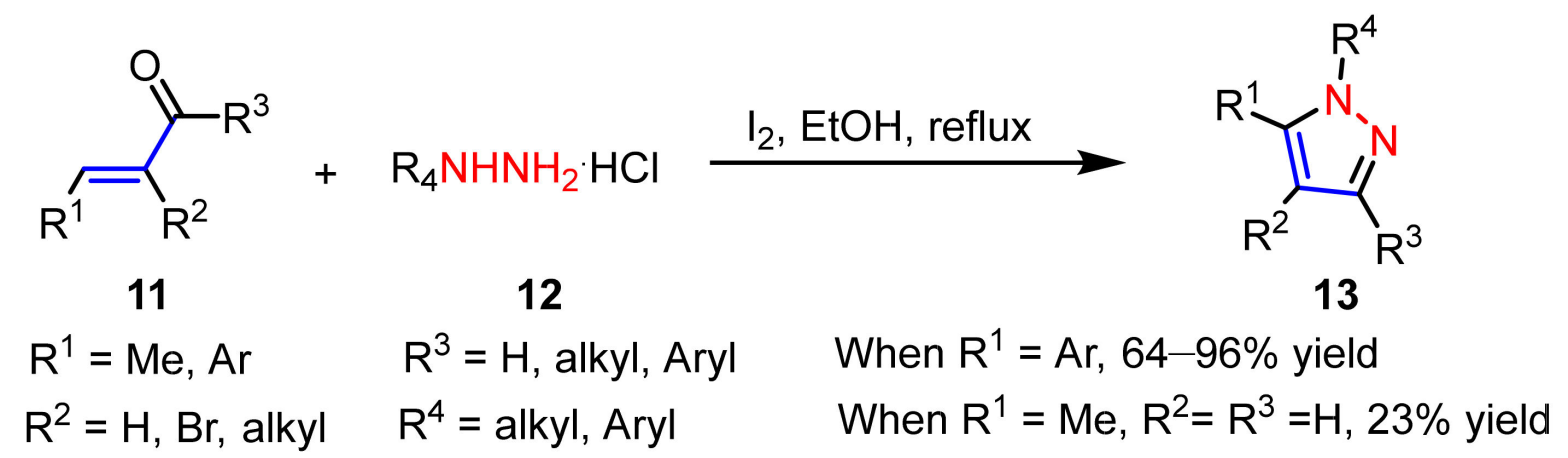
I_2_-mediated cyclocondensation for pyrazole synthesis.

The condensation of hydrazines with α-enones affords pyrazolines, and can be followed by oxidization to generate the corresponding pyrazoles. Zhang et al. reported using iodine to mediate oxidative intramolecular C-N bond formation, and the hydrazone intermediates were cyclized to generate pyrazoles [12]. This one-pot applicable procedure led directly to a variety of di-, tri-, and tetra-substituted (aryl, alkyl, and/or vinyl) pyrazole derivatives with high (64–96%) yields when using β-aryl substituted aldehydes and ketones as substrates. However, the yield decreased sharply when R^1^ was a methyl group (23% yield) due to the loss of the extended conjugation to facilitate the nucleophilic attack of the α,β-unsaturated double-bond to iodine (Scheme 7).

**Scheme 8:**
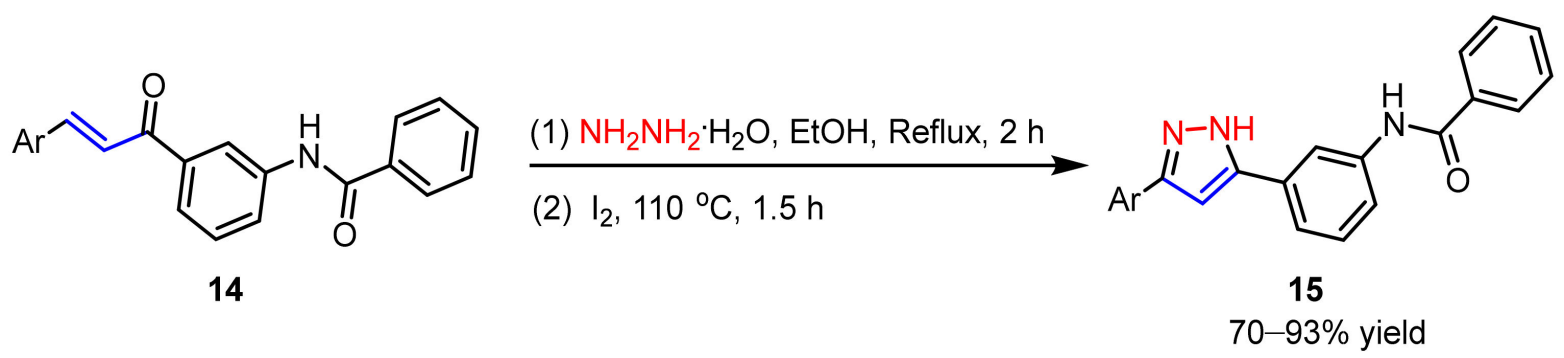
3,5-diarylpyrazole synthesis through I_2_-mediated cyclocondensation.

The applicability of this general method has been extended to synthesize an array of novel 3,5-diarylpyrazole derivatives (**15**); some of these are potent acetylcholinesterase inhibitors possessing excellent selectivity, and have been accepted as prospective drug candidates for treating Alzheimer’s disease (Scheme 8) [13].

**Scheme 9:**
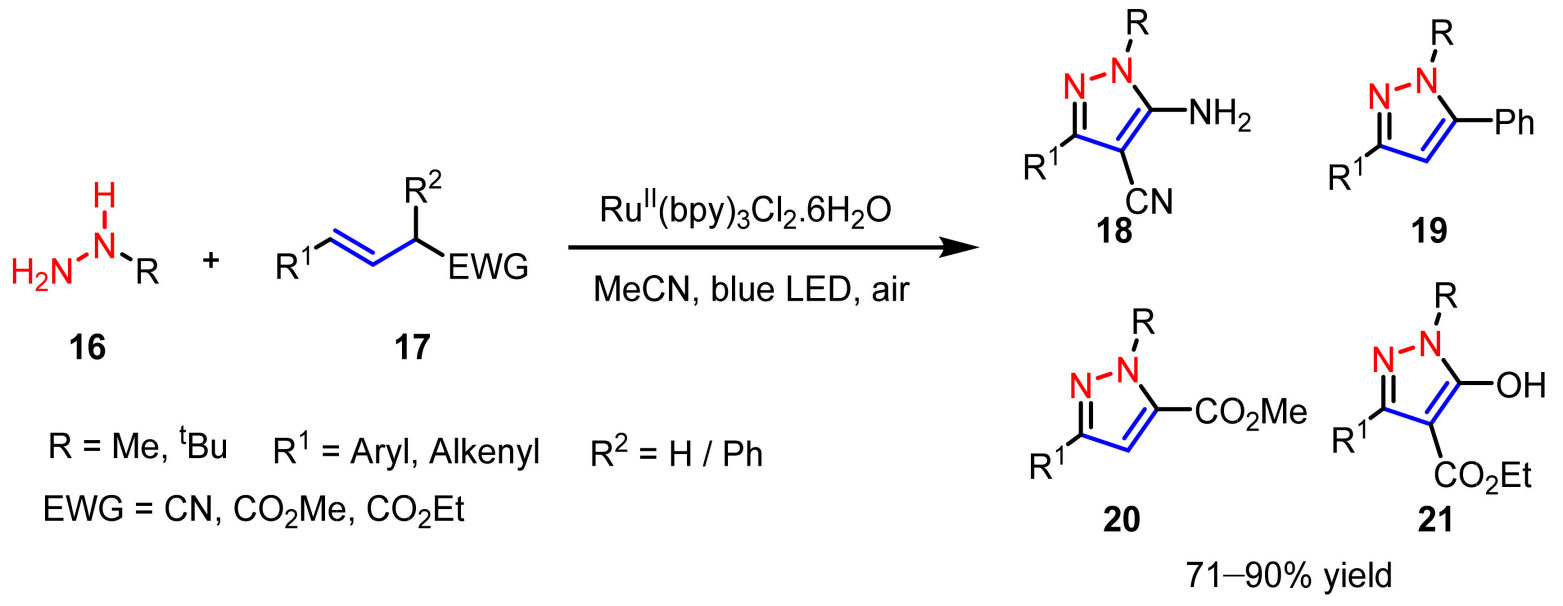
Ru^II^-catalyzed photoredox cyclization for pyrazole synthesis.

In addition, Ding et al. reported an air-promoted photoredox cyclization of substituted hydrazines with activated alkene (Michael addition reaction acceptors) to afford corresponding pyrazoles with good to excellent yields [14]. In this context, hydrazine was oxidized by Ru^II^ to a diazene intermediate that attacks Michael acceptors, followed by an intramolecular cyclization to form pyrazoles. The reduced Ru^I^ was reoxidized by air to recycle this photoredox process. For electron withdrawing groups (EWG) such as cyano or carboxylic acidic groups, this procedure efficiently generated 5-amino and 5-hydroxyl substituted pyrazoles, which are important functionalities for subsequent transformations (Scheme 9).

**Scheme 10:**
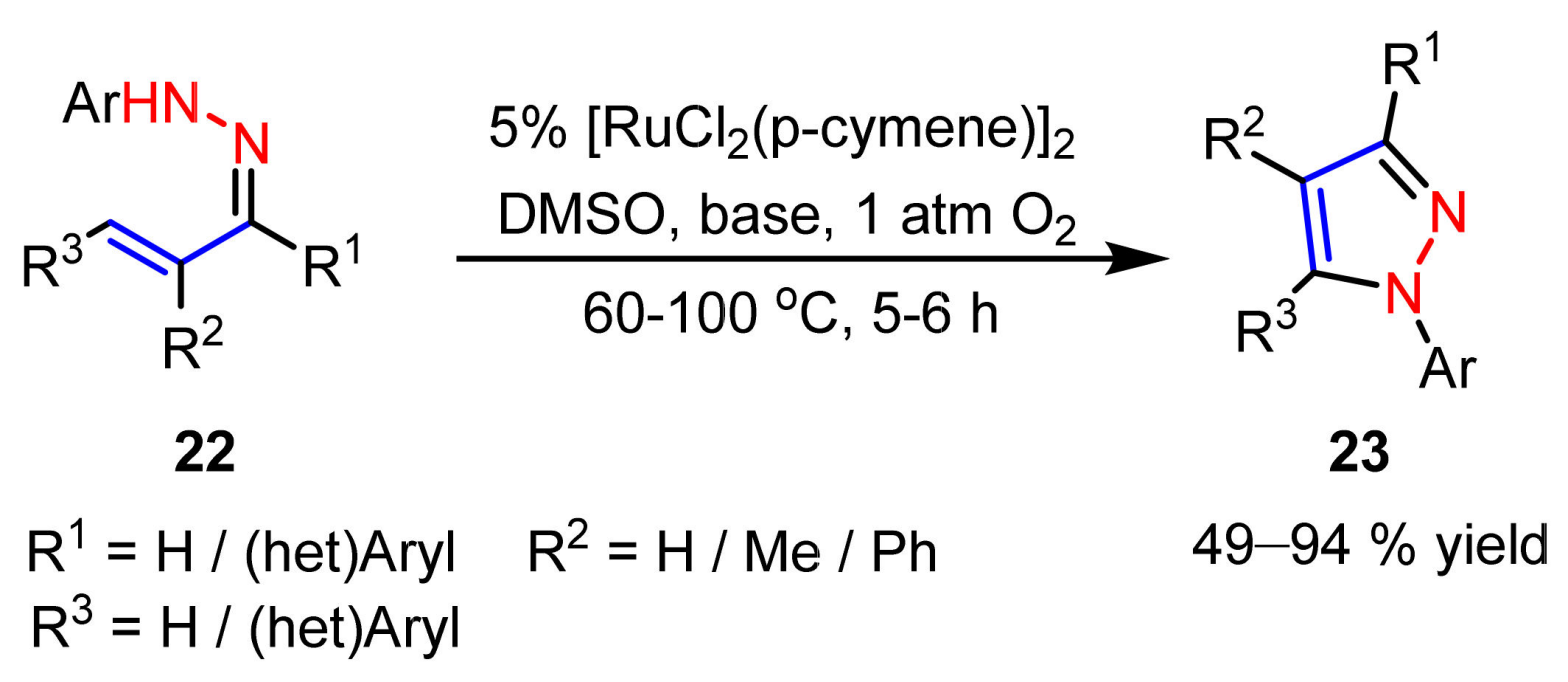
Ru^II^-catalyzed oxidative cyclization for pyrazoles synthesis.

Pre-installation of enones with hydrazines to form hydrazone intermediates is also accessible. Hu and co-workers [15] reported a Ru^II^-catalyzed intramolecular oxidative C–N coupling for the facile synthesis of highly diversified tri- and tetrasubstituted pyrazoles. Their method is applicable to a broad scope of substrates with excellent functional group tolerance. More importantly, many of the pyrazoles generated using this strategy were difficult to prepare by conventional methods (Scheme 10).

#### 2.1.3. Cyclocondensation of Hydrazines with Ynones and Related Compounds

**Scheme 11:**
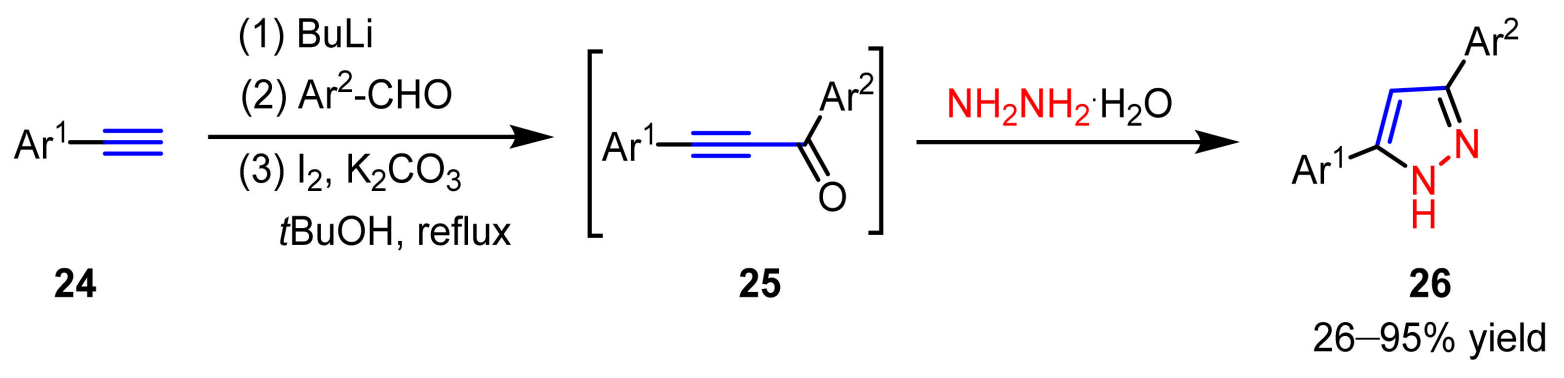
Multicomponent synthesis of pyrazoles from alkynes.

Harigae et al. reported using a one-pot regioselective procedure for the synthesis of 3,5-disubstituted pyrazoles with good yields [16]. In his multicomponent procedure, ynone intermediates were formed through iodine-mediated in situ oxidation of propargylic alcohols that were converted from terminal alkynes and aldehydes (Scheme 11). 

**Scheme 12:**
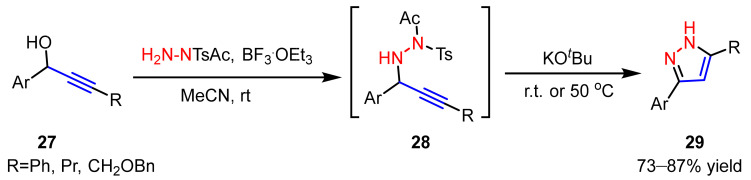
Propargylic alcohols as substrates for two-step pyrazole synthesis.

In addition, propargylic alcohols, the reduced form of ynones, were also used to generate 3,5-disubstituted 1*H*-pyrazoles [17]. This procedure includes two consecutive steps: (1) Lewis acid catalyzed *N*-propargylation of propargylic alcohols to propargyl hydrazides with *N*-acetyl-*N*-tosylhydrazine, followed by (2) base-mediated intramolecular cyclization (Scheme 12).

#### 2.1.4. Cyclocondensation of Hydrazines with Vinyl Ketones Bearing a Leaving Group

**Scheme 13:**
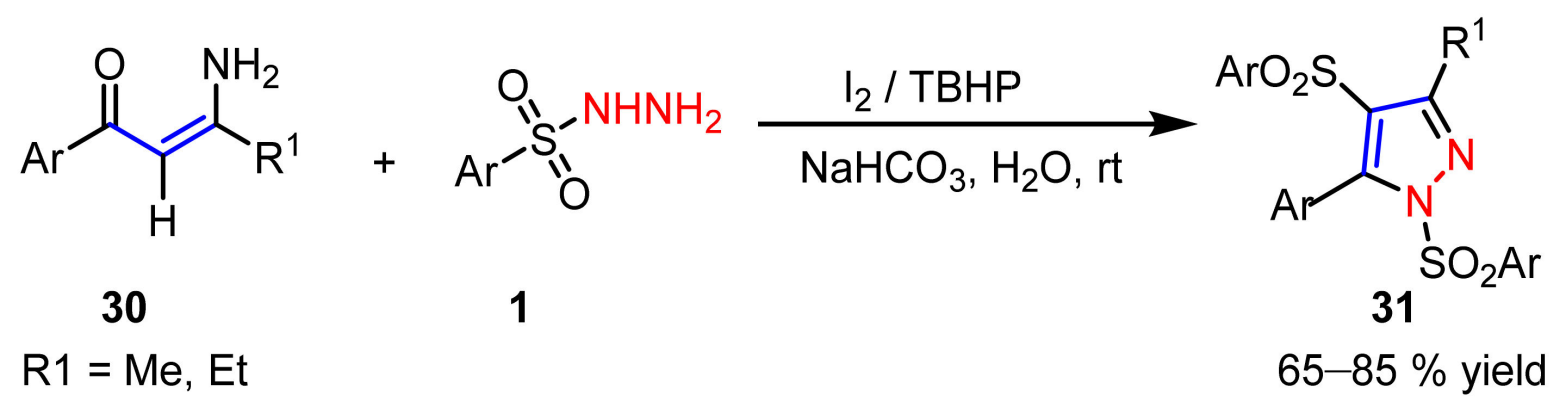
Pyrazole synthesis in water.

Aminomethylene and (dimethyl)aminomethylene groups are synthetic equivalents of a formyl group. α,β-Vinyl ketones containing these substituents may react with hydrazine derivatives to afford pyrazolines, followed by elimination of the leaving group to generate the desired pyrazoles. The methylthio group has also been reported as an excellent leaving group [10]. Guo et al. reported that in the presence of iodine and *tert*-butyl hydroperoxide (TBHP), β-amino vinyl ketone could cyclize with tosyl hydrazine in water to afford fully substituted pyrazoles [18]. Mechanism studies using ^15^N-labeled enaminone as a substrate showed that the β-amino group did not participate in the formation of a pyrazole ring, instead, it acted as a leaving group, serving as a hydrogen bond donor to facilitate the reaction in water (Scheme 13).

**Scheme 14:**
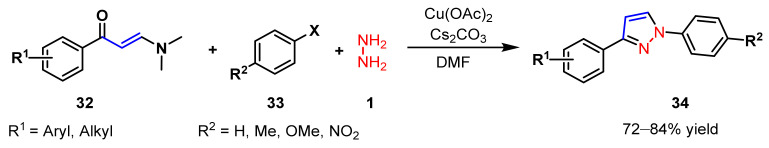
Cu-catalyzed three-component cyclocondensation for pyrazole synthesis.

Raghunadh and co-workers reported a copper-catalyzed three-component process for pyrazole synthesis [19]. The authors found that *N*-substituted pyrazoles could be afforded in high yields and excellent regioselectivity when using β-dimethylamino vinyl ketones as substrates in the presence of aryl halides (Scheme 14).

**Scheme 15:**
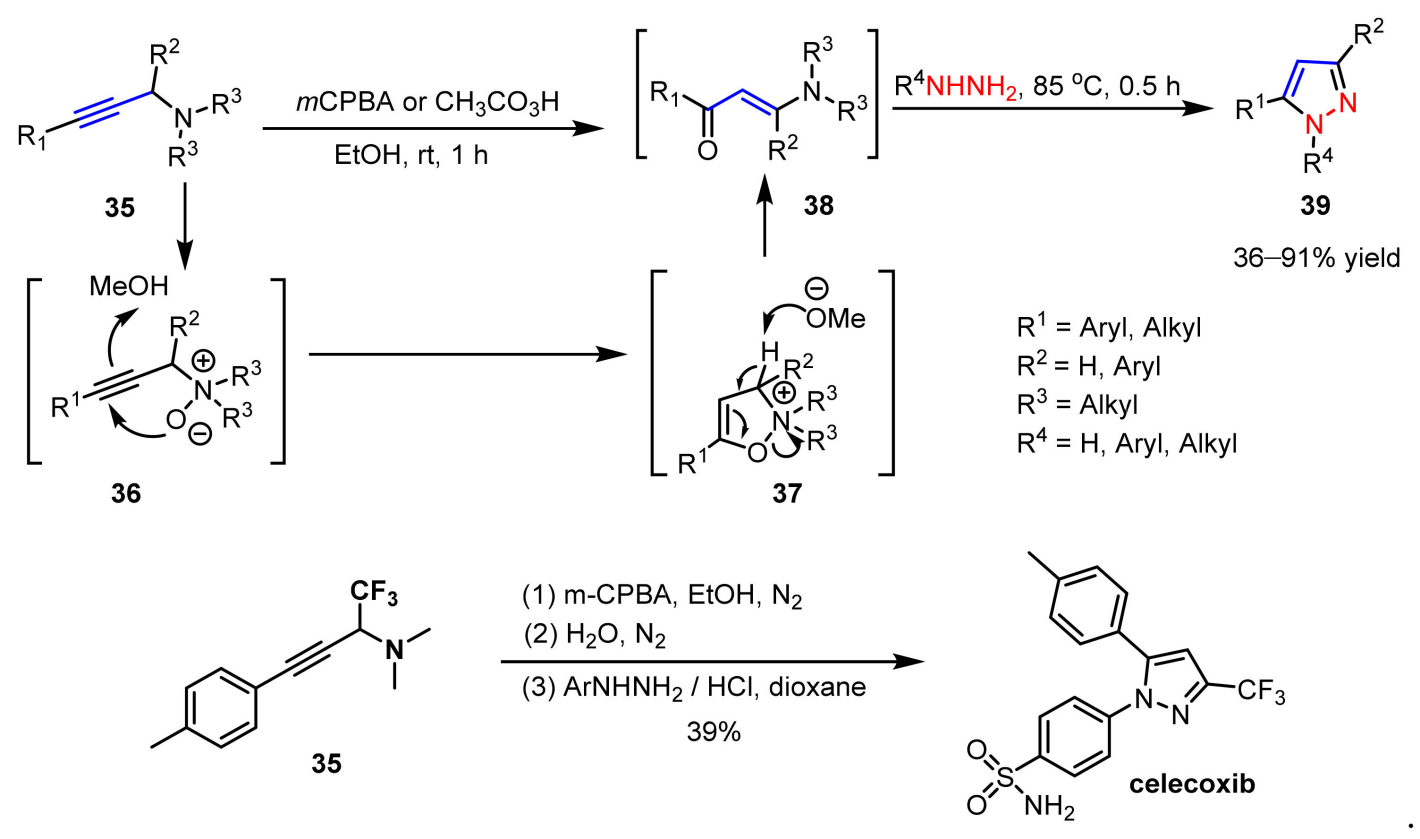
One-pot oxidative cyclocondensation for pyrazole derivatives synthesis.

In the presence of peroxide (*m*-CPBA), Chen et al. showed that propargylamines could be converted into enaminones after a one-pot oxidation and rearrangement process. The formed enaminones are key intermediates that would react with hydrazines to afford corresponding pyrazole derivatives [20]. Using this methodology, the researchers realized a one-pot, four-step celecoxib synthesis with a 39% overall yield starting from 4-ethynyltoluene (Scheme 15).

**Scheme 16:**
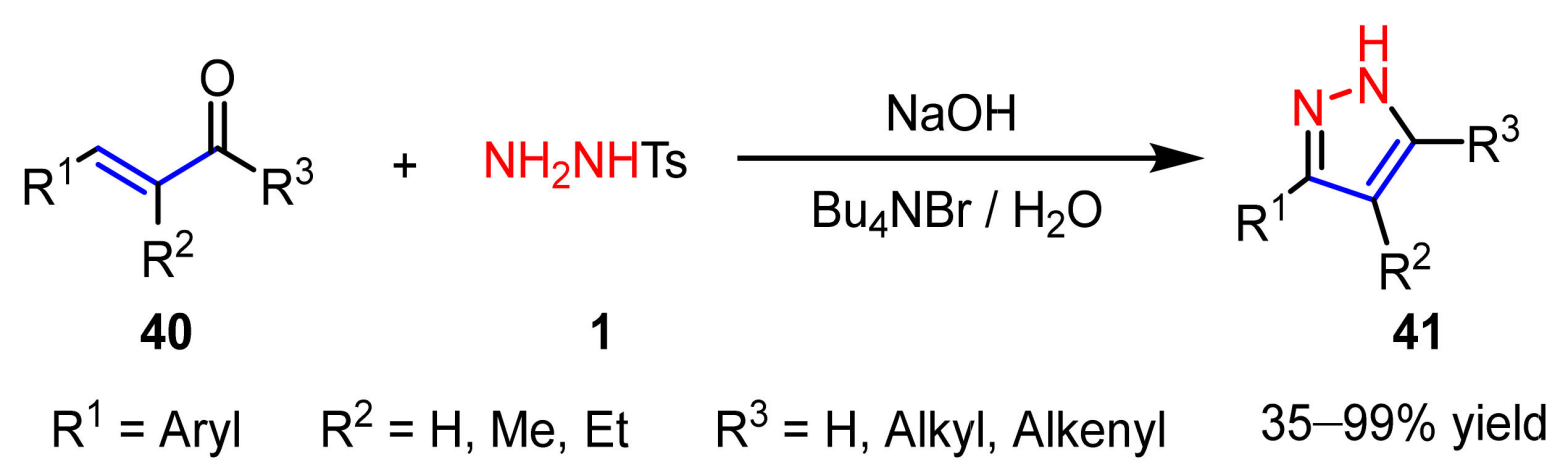
Proton leaving groups in cyclocondensation for pyrazole synthesis.

Under alkaline conditions, a proton on the β-vinyl substrate can serve as a leaving group to accelerate the conversion of pyrazolines to pyrazoles [21]. In addition, when the phase transfer catalyst Bu_4_NBr is used, these reactions can be performed smoothly in water [22] (Scheme 16).

### 2.2. 1,3-Dipolar Cycloadditions

**Scheme 17:**
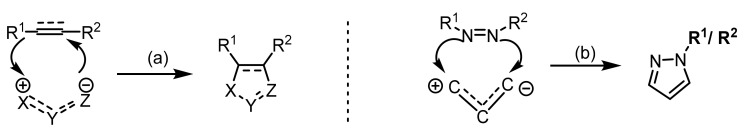
Strategies for 1,3-dipolar cycloaddition. (**a**): [*CNN*] + [*CC*] mode; (**b**): [*CCC*] + [*NN*] mode.

The intrinsically high regioselectivity and efficiency of 1,3-dipolar cycloaddition has led to its prominent role in preparing substituted pyrazoles [5]. Conventional 1,3-dipolar cycloaddition employs a [*CNN*] fragment and a [*CC*] fragment. In general, three main classes of 1,3-dipoles have been used as the [*CNN*] fragment, namely, diazoalkanes, nitrilimines, and azomethine imines, while their [*CC*] counterparts are alkenes or alkynes (Scheme 17a). Additionally, the combination of a [*CCC*] fragment plus a [*NN*] fragment has also been utilized. In these examples, [*CCC*] comes from alkenes or alkynes, while the [*NN*] fragment derives from azo compounds (Scheme 17b).

In this section, we discuss the synthetic development of 1,3-dipolar cycloaddition reactions according to the source of 1,3-dipoles fragments, namely, from diazoalkanes, nitrilimines, or azomethine imine, respectively. Reactions in which azo compounds act as [*NN*] fragments are also presented.

#### 2.2.1. Diazoalkanes as 1,3-Dipoles

**Scheme 18:**
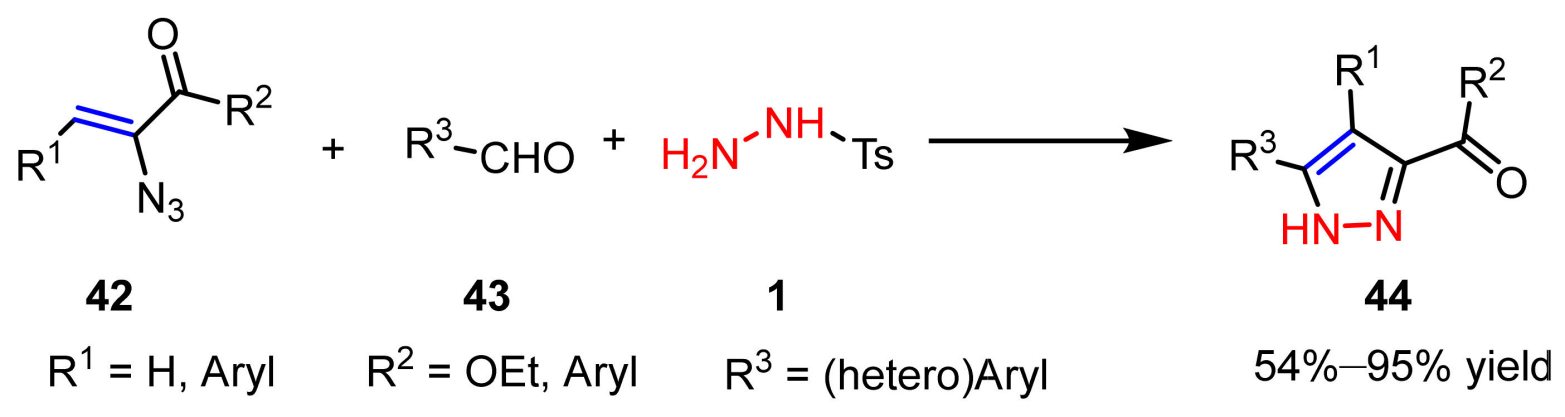
Azide as leaving groups in diazoalkanes for pyrazole synthesis.

Electron-rich diazo compounds are toxic and potentially explosive, thus the preparation and handling of these reactants are hazardous. To overcome this problem, an improved method for making aryldiazomethanes from stable tosylhydrazones derivatives was developed to synthesize pyrazoles. Using an operationally simple, multicomponent, one-pot procedure, 3,4,5-trisubstituted 1*H*-pyrazoles could be prepared from vinyl azide, aldehyde, and tosylhydrazine [23]. In these cases, the azide group serves as a leaving group (Scheme 18).

**Scheme 19:**
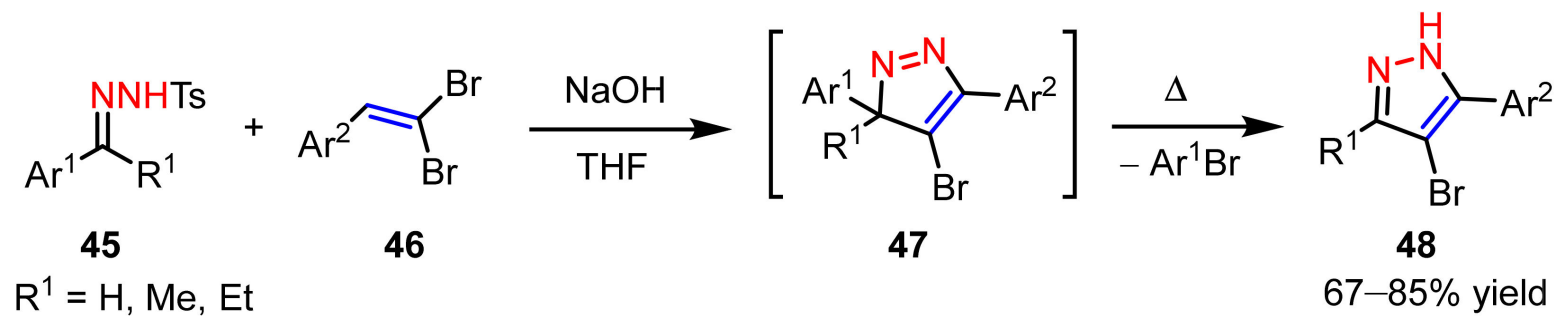
Bromine as the leaving group in diazoalkanes for pyrazole synthesis.

Bromine is also a good leaving group. Sha et al. developed a simple, highly efficient, and regioselective method for the synthesis of 3,5-diaryl-4-bromopyrazoles using *gem*-dibromoalkene as the substrate [24] (Scheme 19).

**Scheme 20:**
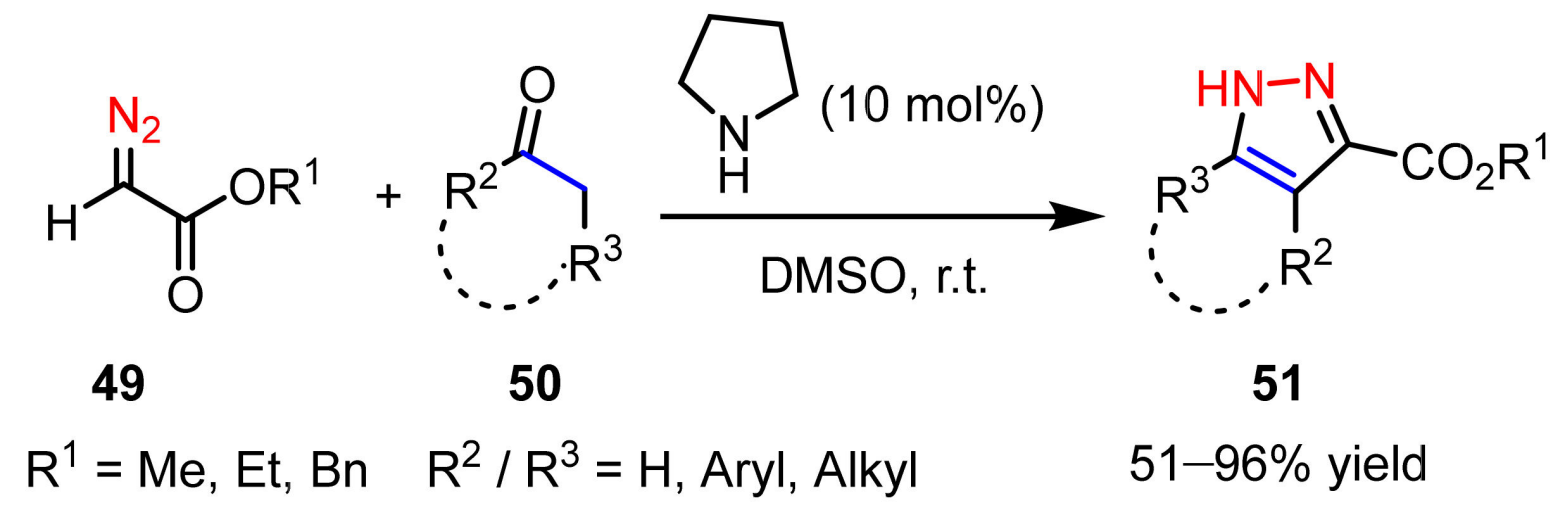
Ketones as substrates in 1,3-dipolar cycloaddition for pyrazole synthesis.

Using pyrrolidine as a catalyst, 3,4,5-trisubstituted or 3,5-disubstituted pyrazoles could be synthesized from carbonyl compounds through an enamine intermediate under mild conditions [25]. Considering that carbonyl compounds are more readily available than alkenes, this optimization is a remarkable advancement. In addition, bicyclic pyrazoles that are difficult to synthesize by common methods can be prepared straightforwardly using this method (Scheme 20).

**Scheme 21:**
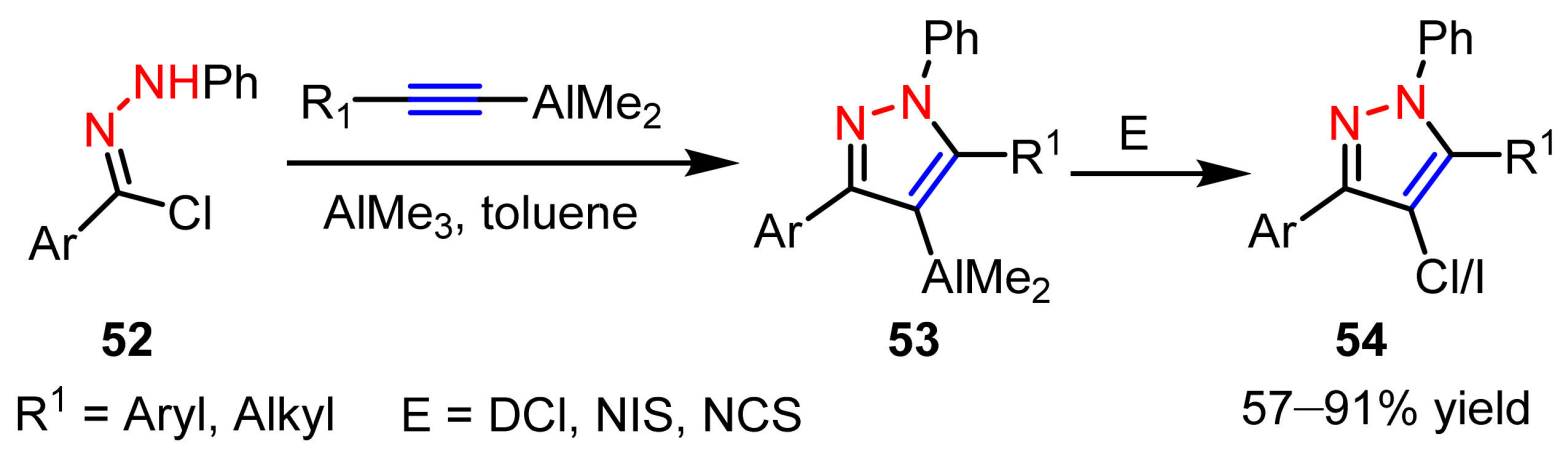
Trimethylaluminum substituted alkynes as substrates for pyrazoles synthesis.

Jackowski et al. reported a novel method to synthesize organoaluminum heterocyclics via a [3 + 2] cycloaddition route [26]. Using this methodology, 3,4,5-trisubstituted pyrazoles could be prepared from reactive polysubstituted alumino-heteroles intermediates (**53**) after a one-step electrophilic substitution (Scheme 21).

#### 2.2.2. Nitrilimines as 1,3-Dipoles

**Scheme 22:**
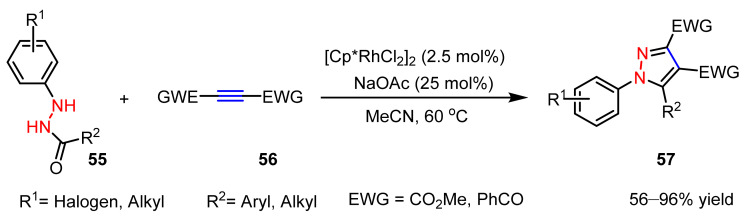
Ru^II^-catalyzed 1,3-dipolar cycloaddition for pyrazole synthesis.

Li and co-workers [27] reported a rhodium-catalyzed cycloaddition of hydrazines with dicarboxylic alkynes. This method provides a highly efficient and effective procedure to synthesize 3, 4-dicarboxylic pyrazoles, and offering versatility for subsequent structural modifications (Scheme 22).

**Scheme 23:**
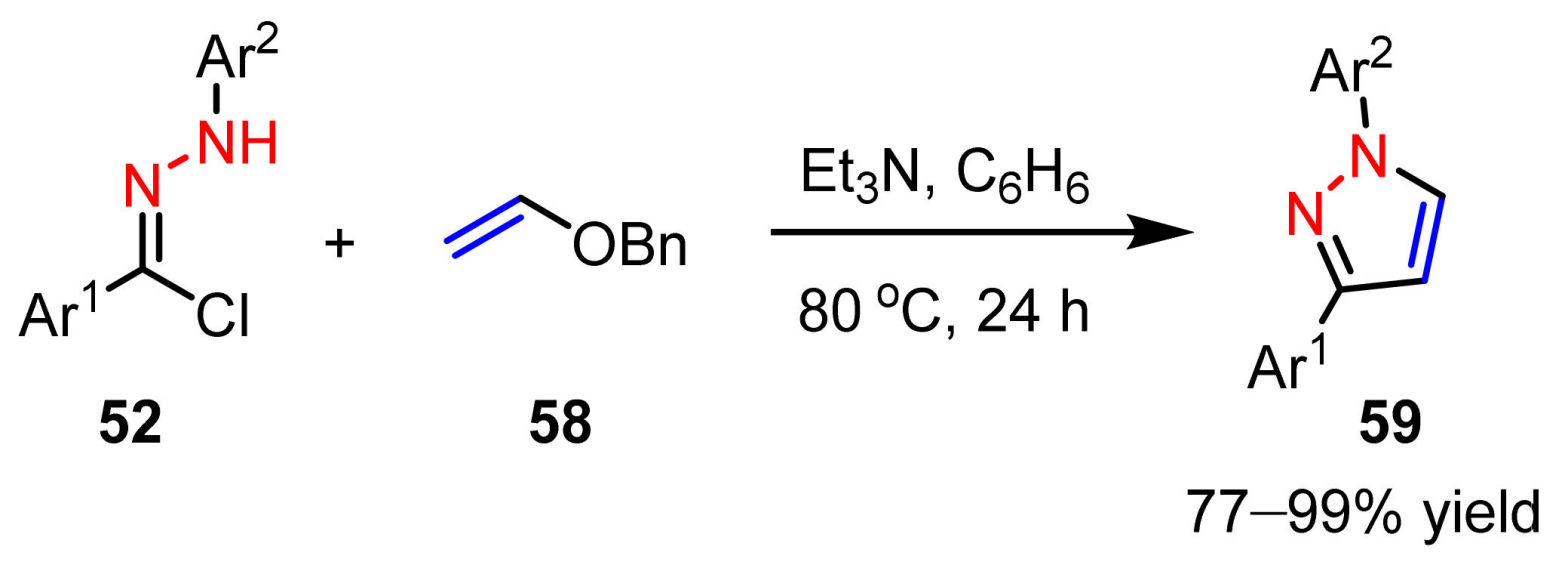
Trimethylamine (TEA) promoted 1,3-dipolar cycloaddition for pyrazoles synthesis.

Ledovskaya et al. reported that the weak organic base trimethylamine (TEA) could promote the 1,3-dipolar cycloaddition of vinyl ethers and hydrazonoyl chlorides to produce 1,3-disubstituted pyrazoles with absolute regioselectivity [28] (Scheme 23).

**Scheme 24:**
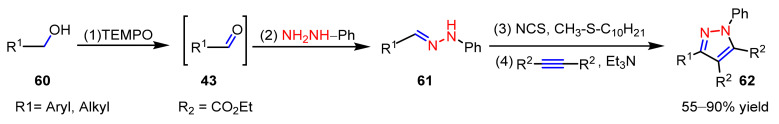
Primary alcohols as substrates for pyrazole synthesis.

Starting from primary alcohols, Kobayashi et al. reported a one-pot, multicomponent procedure for making multisubstituted pyrazoles [29]. According to their description, the primary alcohol was first oxidized by 2,2,6,6-tetramethylpiperidinyloxy (TEMPO) to aldehyde, followed by reacting with hydrazine to form oxime, where the latter was converted into nitrilimine in the presence of *N*-chlorosuccinimide (NCS) and decyl methyl sulfide, followed by a 1,3-dipolar cycloaddition between nitrilimine and diethyl acetylenedicarboxylate (Scheme 24).

**Scheme 25:**
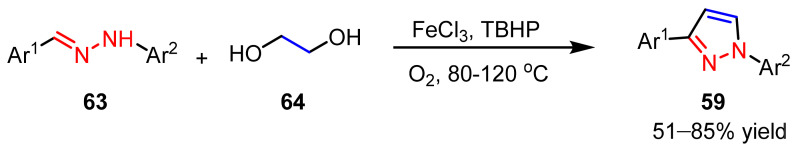
Fe^III^-catalyzed 1,3-dipolar cycloaddition for pyrazole synthesis.

Using alcohols as potential [*CC*] fragments, Panda et al. reported an iron-catalyzed route [30] for the regioselective synthesis of 1,3- and 1,3,5-substituted pyrazoles by the condensation of diarylhydrazones with α-carbonyl alcohols, which were converted in situ from vicinal diols by ferric chloride in the presence of *tert*-butyl hydroperoxide (TBHP) (Scheme 25).

**Scheme 26:**
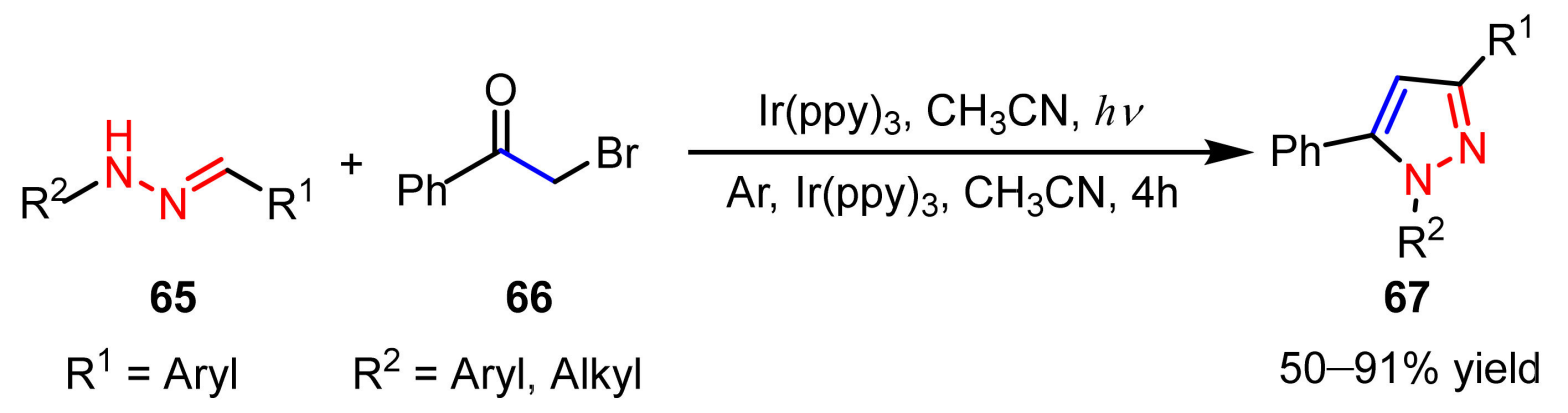
Ir^III^-catalyzed photoredox cycloaddition for pyrazole synthesis.

A one-pot visible light-promoted single-electron-transfer (SET) process for making 1,3,5-trisubstituted pyrazoles from α-bromoketones was reported by Fan and co-workers [31]. The reaction has good functional group tolerance, even for nitro and nitrile, which are generally not tolerated in SET reactions (Scheme 26).

**Scheme 27:**
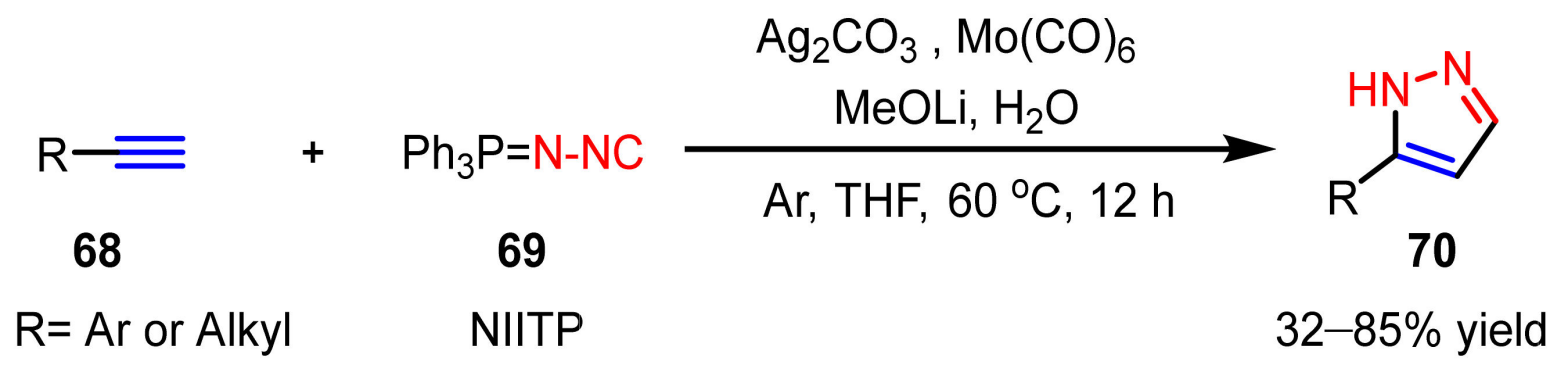
Ag^II^-mediated cycloaddition for pyrazole synthesis.

Yi et al. reported a novel silver-mediated [3 + 2] cycloaddition of alkynes and *N*-isocyanoiminotriphenylphosphorane (NIITP) for the assembly of monosubstituted pyrazoles [32]. The reaction can be accomplished under mild conditions, with broad substrate scope and excellent functional group tolerance. Mechanism studies showed that NIITP was activated by Mo(CO)_6_, then undergoes a [3 + 2] cycloaddition with a silver acetylide intermediate (Scheme 27). 

**Figure 3:**
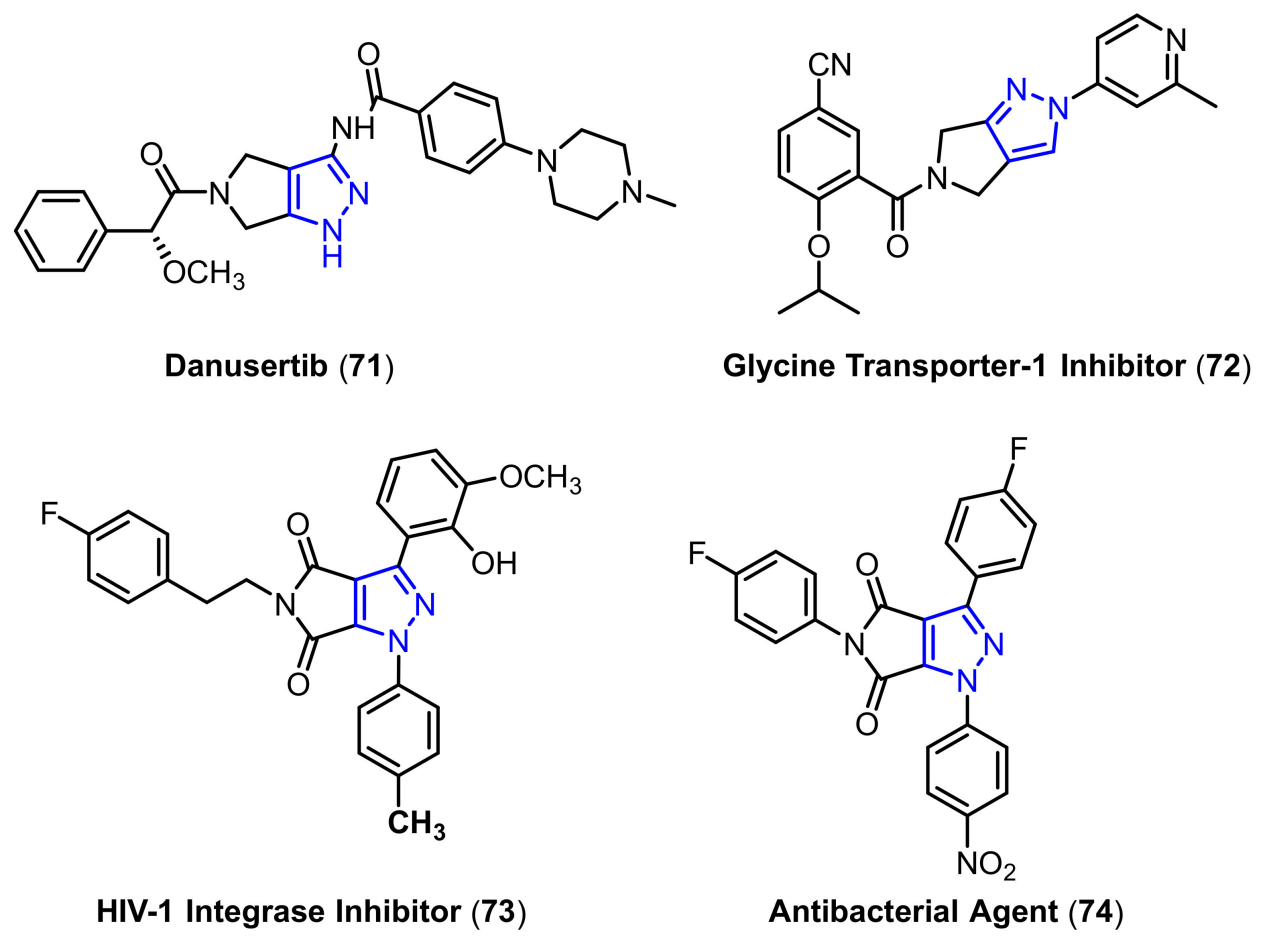
Bioactive molecules containing pyrrolopyrazole motifs.

Bioactive molecules with pyrrolopyrazole motifs such as an aurora kinase inhibitor danusertib (**71**), a glycine transporter-1 inhibitor (**72**), an HIV-1 integrase inhibitor (**73**), and an antibacterial agent (**74**) are promising drug candidates [33] (Figure 3). Therefore, general and practical methods for making such compounds are valuable.

**Scheme 28:**
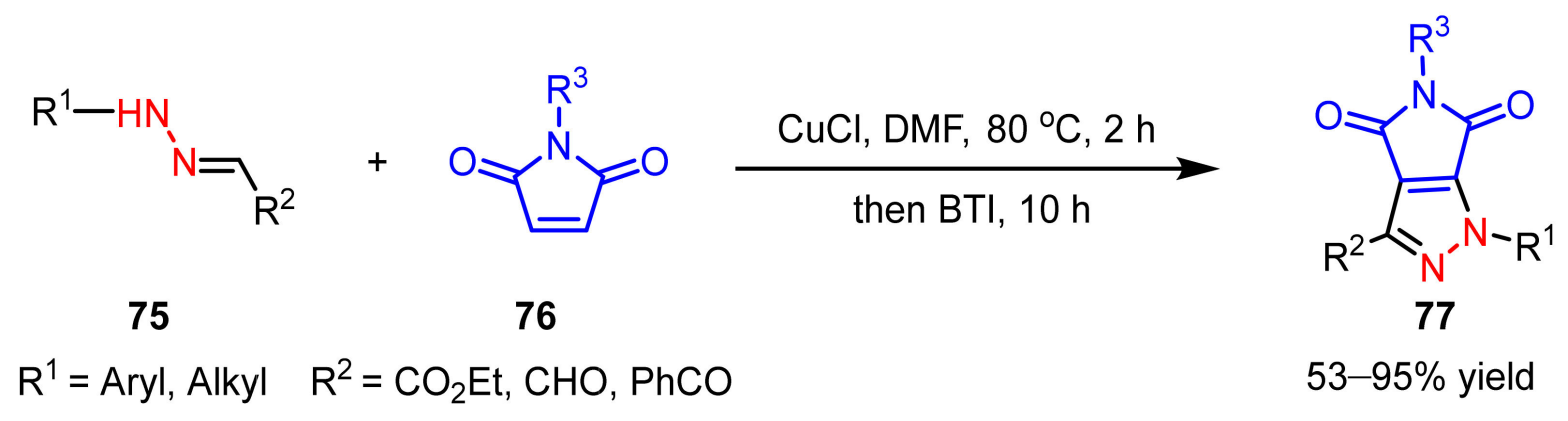
Cu^Ⅰ^-catalyzed cycloaddition for pyrrolopyrazole synthesis.

Zhu et al. reported a CuCl-catalyzed oxidative coupling reaction of aldehyde hydrazones with maleimides to prepare dihydropyrazoles under mild conditions [33]. Using this method, a variety of pyrrolo[3,4-*c*]pyrazoles could be obtained from dihydropyrazoles by one-step following oxidation (Scheme 28).

#### 2.2.3. Sydnones as 1,3-Dipoles

**Scheme 29:**
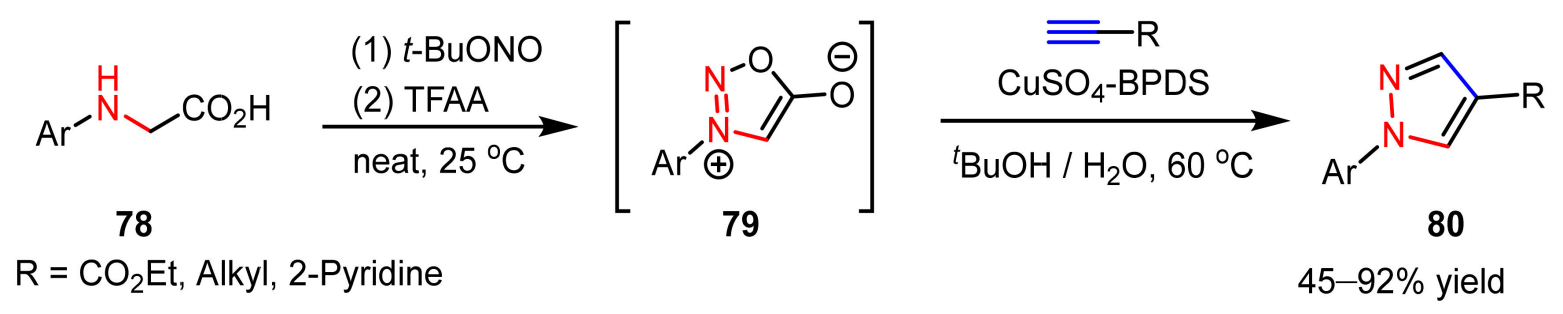
Sydones as 1,3-dipoles for pyrazole synthesis.

Specklin et al. reported a one-pot Cu-catalyzed sydnone-alkyne cycloaddition to generate 1,4-disubstituted pyrazoles from readily available arylglycines [34]. This method tolerates various electron-rich or electron-poor *N*-aryl sydnones, and acetylene components. Using this method, 1,4-pyrazoles were the only products with good to excellent yield; no trace of 1, 3-regioisomers was detected (Scheme 29).

**Scheme 30:**
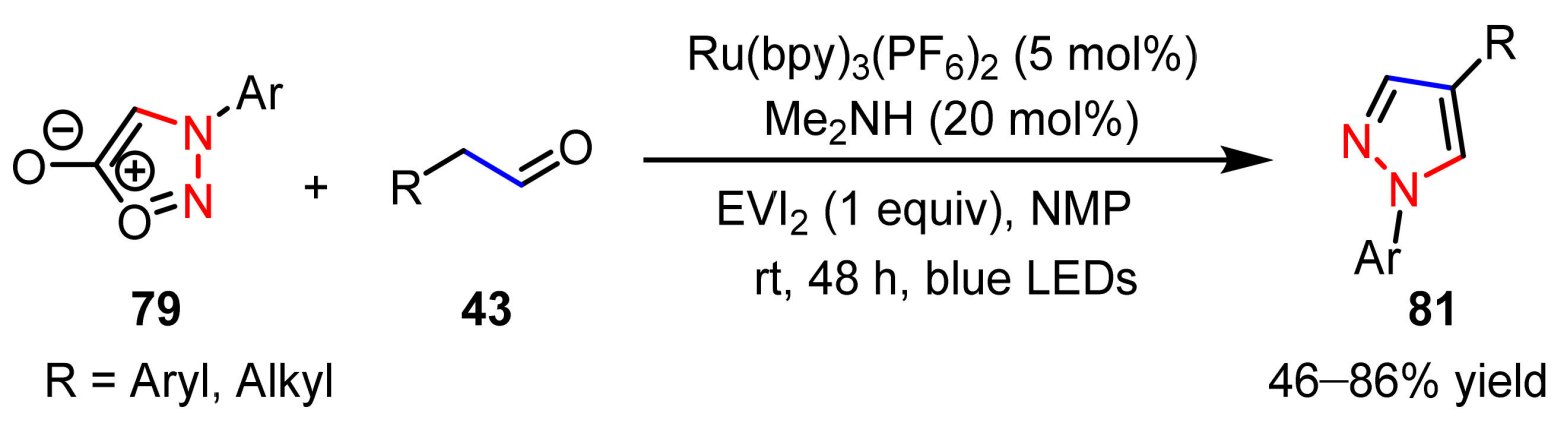
Ru^II^-catalyzed photoredox cycloaddition for pyrazole synthesis.

In addition, a visible-light photoredox process using Ru(bpy)_3_(PF_6_)_2_ as the catalyst to accomplish this regioselective reaction was also reported. In this case, a broad scope of 1,4-disubstituted pyrazoles was made in high yields [35] (Scheme 30).

#### 2.2.4. Azo Compounds as [*NN*] Fragments

**Scheme 31:**
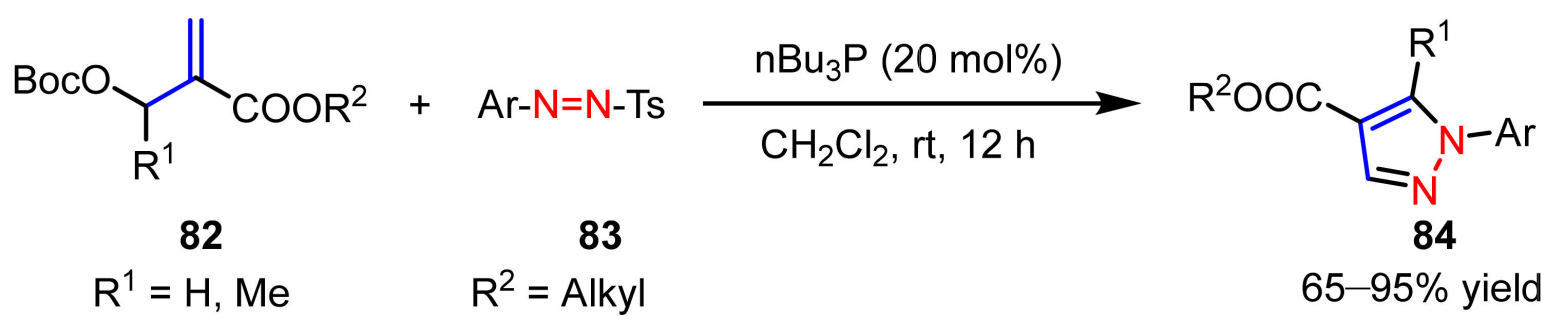
Azo compounds as [*NN*] fragment for pyrazole synthesis.

Zhang et al. reported a highly efficient *n*Bu_3_P-catalyzed desulfonylative [3 + 2] cycloadditions of allylic carbonates with arylazosulfones to make 1,4-disubstitued pyrazoles in good to excellent yields under mild conditions [36]. The reaction can be triggered by the Michael-type addition of *n*Bu_3_P to allylic carbonate, meanwhile, an allylic phosphorus ylide intermediate was formed after decomposition of the Boc group into CO_2_ and *t*BuOH. The ylide intermediate subsequently underwent a [3 + 2] cycloaddition with arylazosulfones to afford the substituted pyrazoles (Scheme 31).

**Scheme 32:**
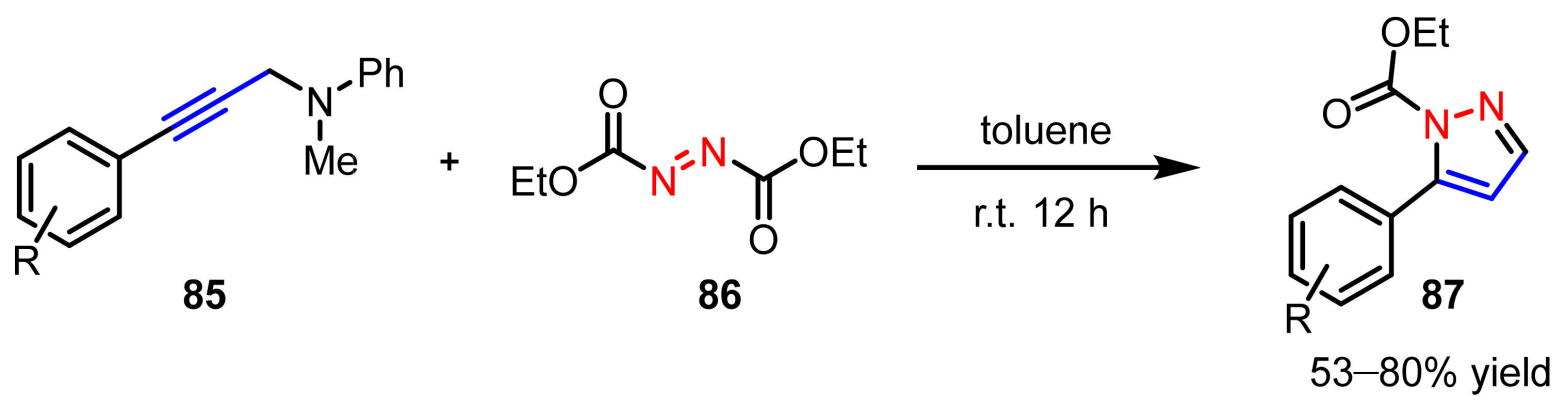
Dialkyl azodicarboxylates (DEAD) as substrates in cycloaddition for pyrazole synthesis.

For some reactions, the involvement of organophosphorus in azo-type cycloadditions is useful, but not essential. Zhang and co-workers [37] reported that substituted propargylamines could react with commercially available dialkyl azodicarboxylates (DEAD) in toluene at room temperature without the presence of organophosphorus. This synthetic strategy was also applicable for the synthesis of different pyrazoles in high yields with broad substrate scopes (Scheme 32).

### 2.3. New Approaches to Reactions for Pyrazole Synthesis

**Scheme 33:**
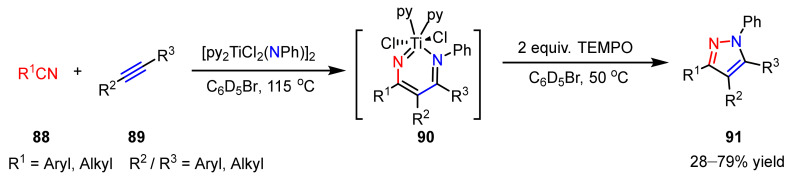
Organotitanium intermediates for pyrazole synthesis.

In each of the above-mentioned methodologies for pyrazole synthesis, all the [*NN*] fragments either came from hydrazine derivatives or from azo compounds. Pearce and co-workers recently reported a novel fragment combination mode [*NC*] + [*CC*] + [*N*] using a multicomponent oxidative coupling to make multi-substituted pyrazoles [38]. In these reactions, diazatitana-cyclohexadiene intermediates that were generated from alkynes, nitriles, and titanium imido complexes, could produce pyrazole derivatives after a 2-electron oxidation process triggered by oxidant TEMPO (Scheme 33).

## 3. The Functionalization of Pyrazoles

### 3.1. The Synthesis of Fluorine-Containing and Fluoroalkyl Substituted Pyrazoles

**Figure 4:**
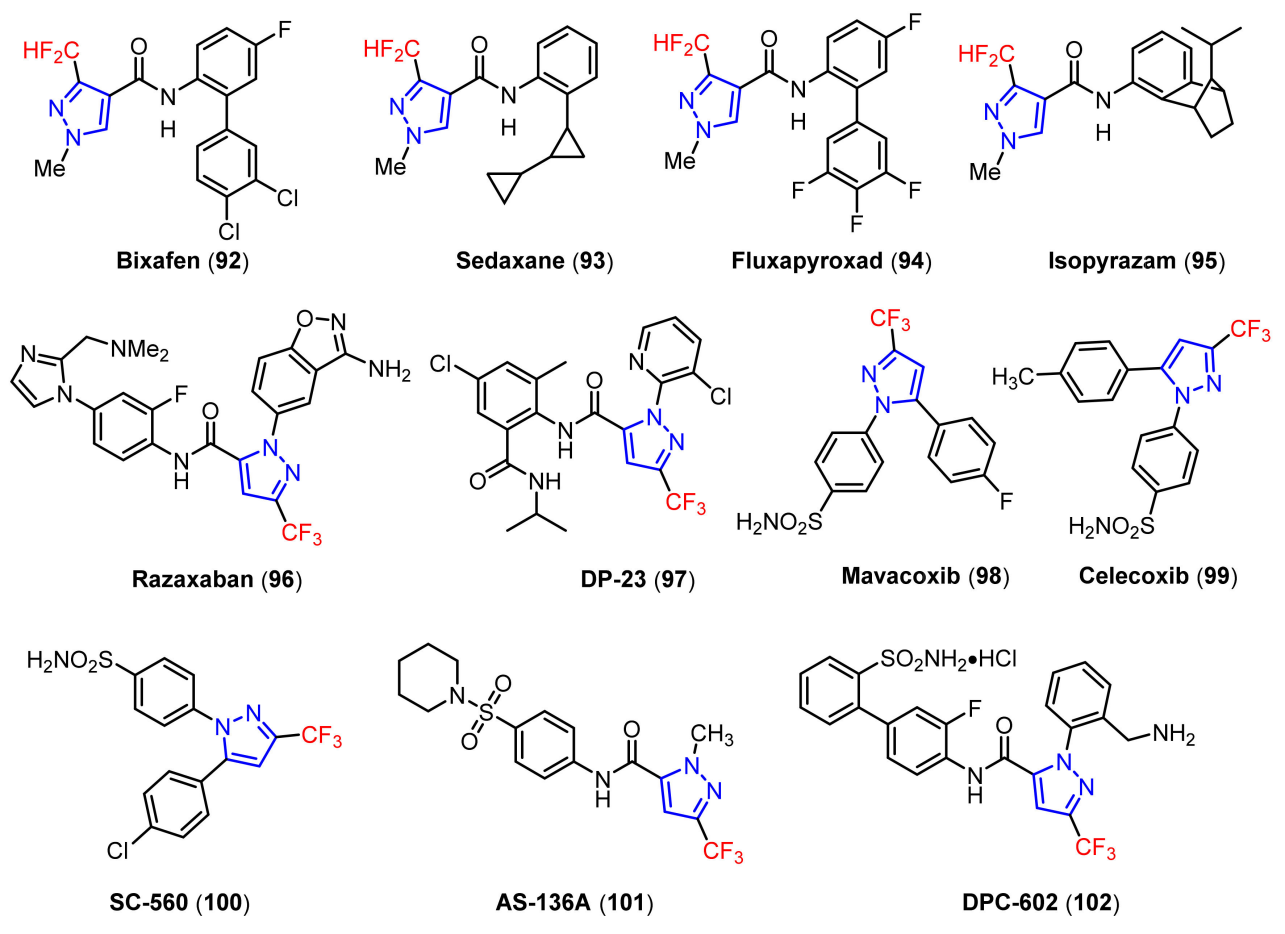
Examples of bioactive molecules with fluorine-containing pyrazole moieties.

The incorporation of fluorine or fluoroalkyl groups onto a pyrazole ring [1,39] can positively affect its physicochemical and biological properties. Exploration of these compounds has led to the successful commercial application of numerous fluorine-containing pyrazoles, as shown in Figure 4. Therefore, new methods of synthesizing fluorine containing pyrazoles may facilitate new drug discoveries, both in pharmaceutical and agrochemical industries. In addition, the incorporation of fluorine in pyrazole motifs can enable ^18^F-labeling for preclinical and clinical position emission tomography (PET) applications. As Gomes et al. have published a recent detailed review on this topic [6], we will not discuss it in this manuscript.

#### 3.1.1. The Synthesis of Monofluorine-Substituted Pyrazoles

**Scheme 34:**
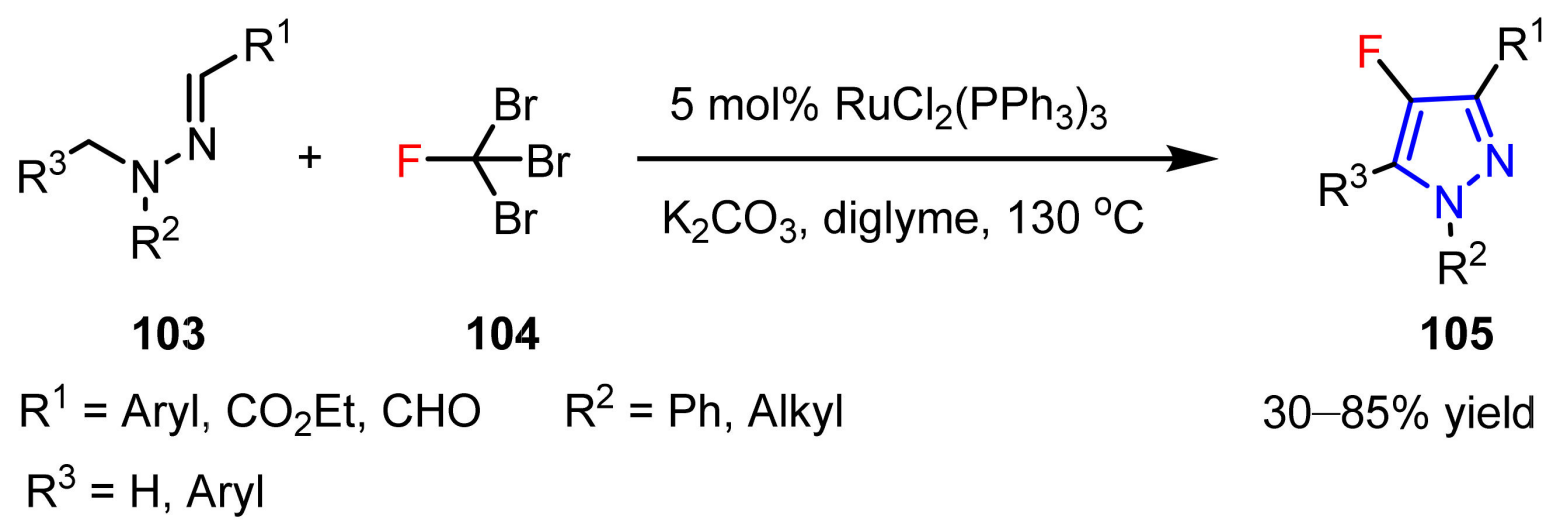
Ru^II^-catalyzed synthesis of monofluorine substituted pyrazoles.

Prieto and co-workers [40] described an accessible method to synthesize 4-fluoropyrazoles by ruthenium-catalyzed tandem C–H fluoromethylation and cyclization of *N*-alkylhydrazones. In this context, CBr_3_F was acting as the fluorine source and a one-carbon unit. Compared to a range of other transition-metal catalysts (Cu, Pd, and Fe), RuCl_2_(PPh_3_)_3_ was the most efficient for this reaction (Scheme 34).

#### 3.1.2. The Synthesis of Difluoromethylpyrazoles

**Figure 5:**
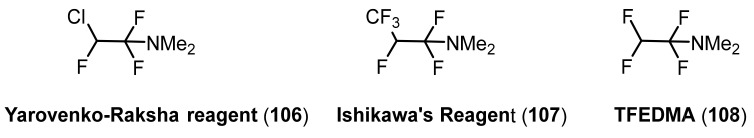
Representative fluoroalkyl amino reagents.

Difluoromethyl-substituted pyrazoles are valuable scaffolds for the preparation of fungicides in agriculture (Figure 4) [1]. Recently, industrial materials like ethyl 2,2-difluoroacetate and fluoroalkyl amino reagents (FAR) (Figure 5) have been widely employed as building blocks for making difluoromethyl pyrazoles [41,42].

**Scheme 35:**
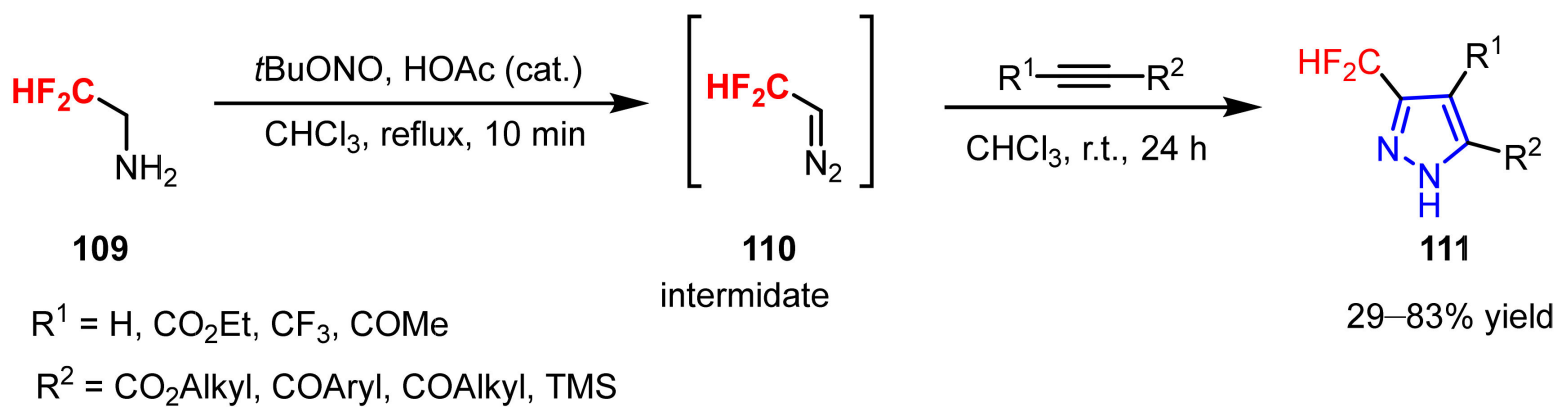
Synthesis of difluoromethyl pyrazoles.

Mykhailiuk [43] reported a novel approach to preparing difluoromethyl-substituted pyrazoles by a [3 + 2] cycloaddition between alkynes and CF_2_HCHN_2_, which was generated in situ from readily available CF_3_CH_2_NH_2_. This practical methodology uses a two-step, one-pot procedure, and does not require either the involvement of a catalyst, or the isolation of a potentially toxic and explosive gaseous intermediate. More importantly, this operationally feasible approach supports scale-up synthesis of target pyrazoles (Scheme 35).

#### 3.1.3. The Synthesis of Trifluoromethyl Pyrazoles

Among fluorinated pyrazoles, the synthesis of 3-trifluoromethyl pyrazoles has attracted significant attention because trifluoromethyl, a strong electronic withdraw group, may increase the bioactivity of target molecules. The feasibility of this tactic is evidenced by the success of a variety of pharmaceuticals and agrochemicals such as razaxaban (**96**, anticoagulant), and DP-23 (**97**, insecticidal activity), mavacoxi (**98**) and celecoxib (**99**) (both are COX-2 inhibitors), and SC-560 (**100**, human lung cancer inhibitor), AS-136A (**101**, measles virus inhibitor), and DPC-602 (**102** arterial thrombosis) (Figure 4). Conventional synthetic methods often suffer from poor regioselectivity between the 3-isomer and 5-isomer when using trifluoromethyl substituted 1,3-dicarbonyl compounds as starting materials [44].

**Scheme 36:**
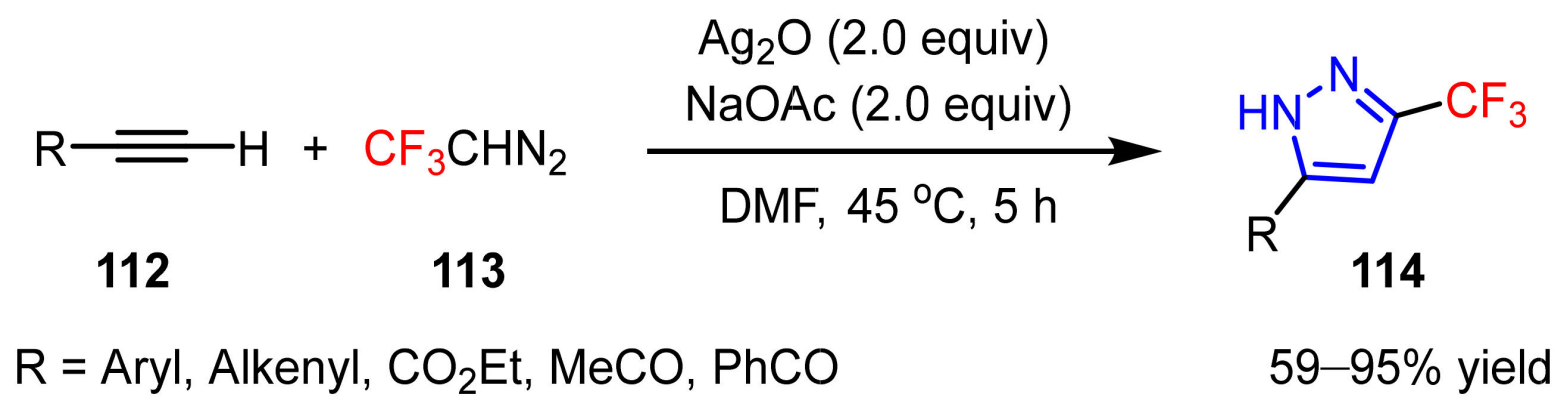
Ag^Ⅰ^-promoted synthesis of 3-trifluoromethyl pyrazoles.

Li et al. reported a highly regioselective approach to the synthesis of 3-trifluoromethyl pyrazoles using silver-mediated cycloaddition of alkynes with 2,2,2-trifluorodiazoethane, which is generated from readily available CF_3_CH_2_NH_2_·HCl [45]. This mild procedure is applicable for the synthesis of celecoxib and measles virus inhibitor AS-136. Interestingly, the reaction only proceeds well in the presence of a trace amount of water (0.1–1 equivalent), otherwise, the yield decreased sharply in the presence of either no water or too much water (>10 equivalent). A deuterated substitution experiment suggested that the proton on 4-position is abstracted from the water medium. Further mechanism studies indicated that silver acetylide might be the active species, as the addition of silver oxide was essential to facilitate this cycloaddition reaction (Scheme 36).

**Scheme 37:**
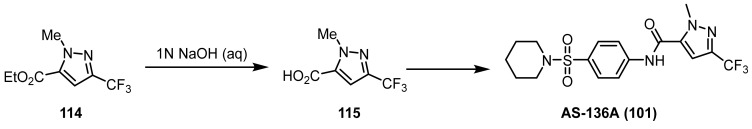
The synthesis of AS-136A from compound **114**.

More importantly, the obtained products through this method are versatile important intermediate chemicals in the pharmaceutical industry. For example, compound **114** is a useful intermediate for the synthesis of measles virus (MV) inhibitor AS-136A (**101**), which displayed nanomolar inhibition against the MV replication in the context of virus infection (Scheme 37) [45,46].

**Scheme 38:**
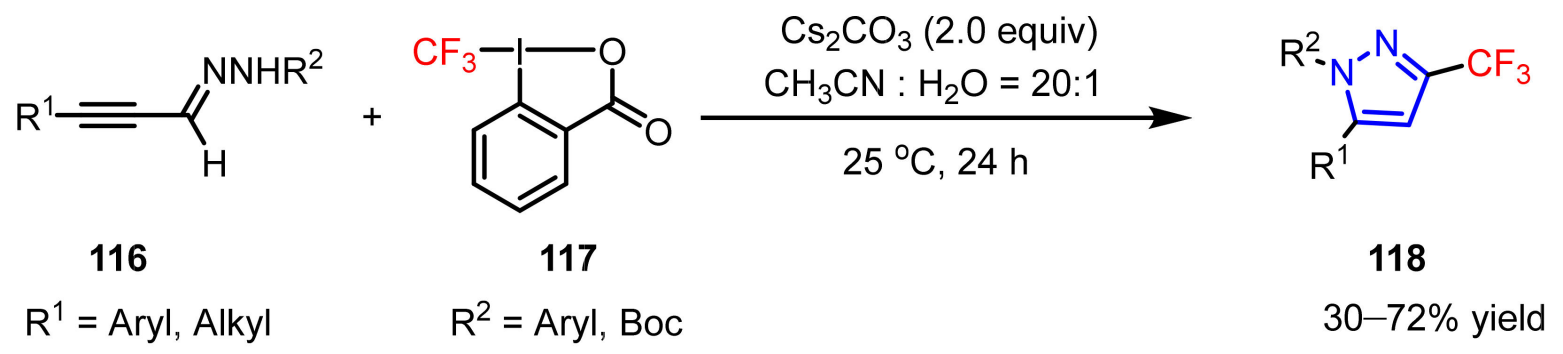
Base-promoted synthesis of 3-trifluoromethyl pyrazoles.

**Scheme 39:**
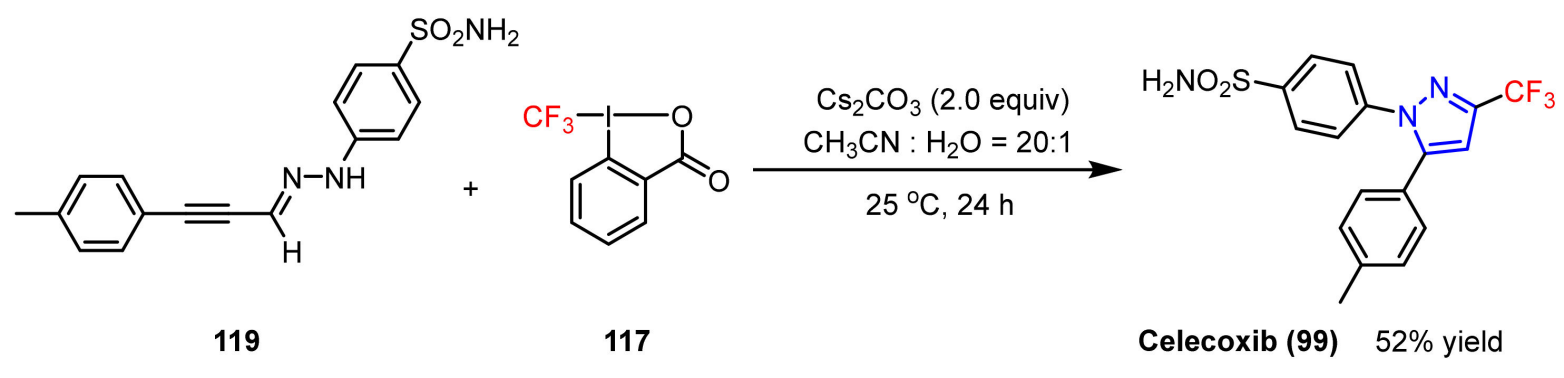
Celecoxib synthesis using a hypervalent iodine reagent.

Subsequently, in 2014, Ji and co-workers [47] reported on an electrophilic trifluoromethylation to synthesize 3-trifluoromethylpyrazoles. This transition-metal free protocol provided a range of 1,3,5-trisubstituted pyrazoles with moderate yield; preliminary mechanism studies indicated that a trifluoromethyl radical species was involved in this reaction (Scheme 38). For example, celecoxib (**99**), a nonsteroidal anti-inflammatory drug, was made using this method with high efficiency and simplicity (Scheme 39) [47].

**Scheme 40:**
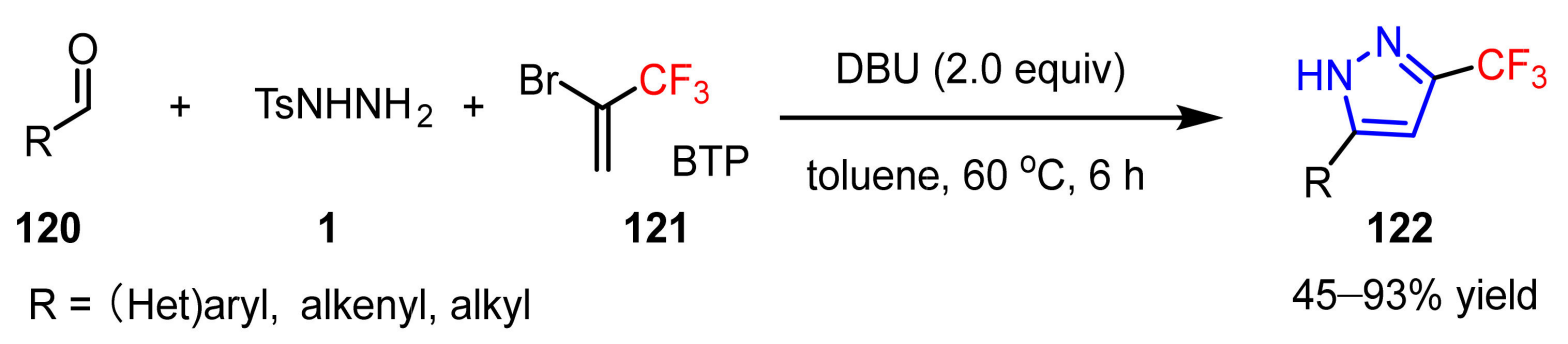
2-bromo-3,3,3-trifluoropropene (BTP) as a building block for the synthesis of 3-trifluoromethylpyrazoles.

Zhu et al. reported a multicomponent method of synthesizing 3-trifluoromethyl pyrazole derivatives through the interactions of readily available 2-bromo-3,3,3-trifluoropropene (BTP), aldehydes, and sulfonyl hydrazides [48]. The protocol features are mild conditions, a broad scope of substrates, high yields, and valuable functional group tolerance. More importantly, this method is highly practical for large-scale production (Scheme 40).

**Scheme 41:**
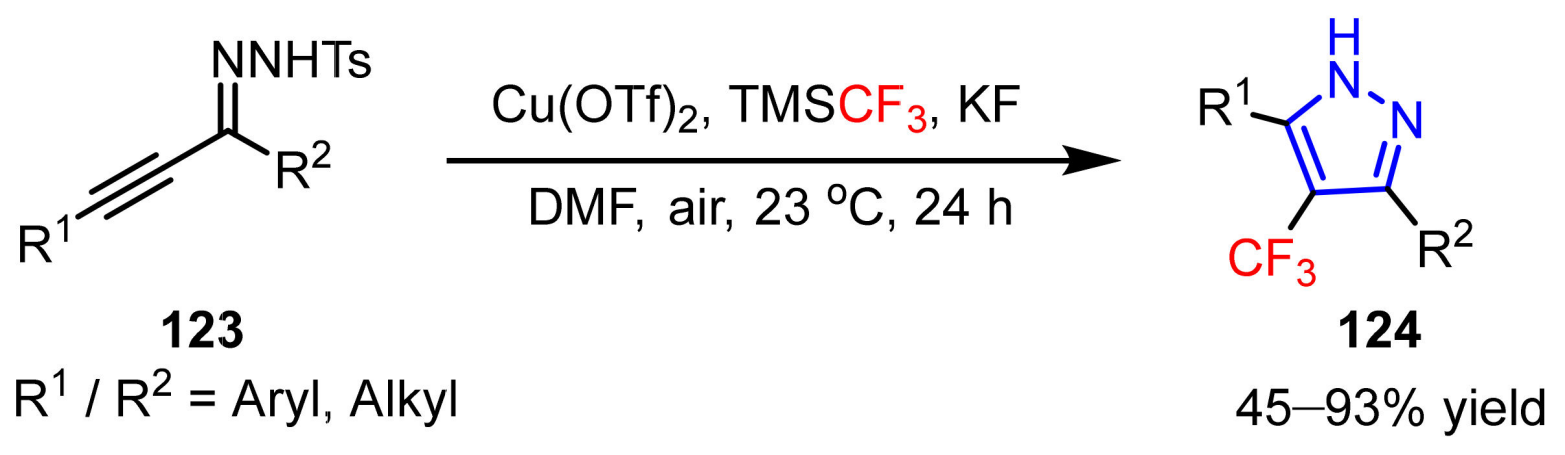
Cu^II^-mediated 4-trifluoromethylpyrazoles.

Wang and co-workers [49] reported a mild method of synthesizing 4-(trifluoromethyl)pyrazoles in high yields; this copper-mediated method, using nucleophilic trifluoromethyltrimethylsilane (TMSCF_3_) as the CF_3_ source, is an important supplement to electrophilic trifluoromethylation reactions (Scheme 41).

**Scheme 42:**
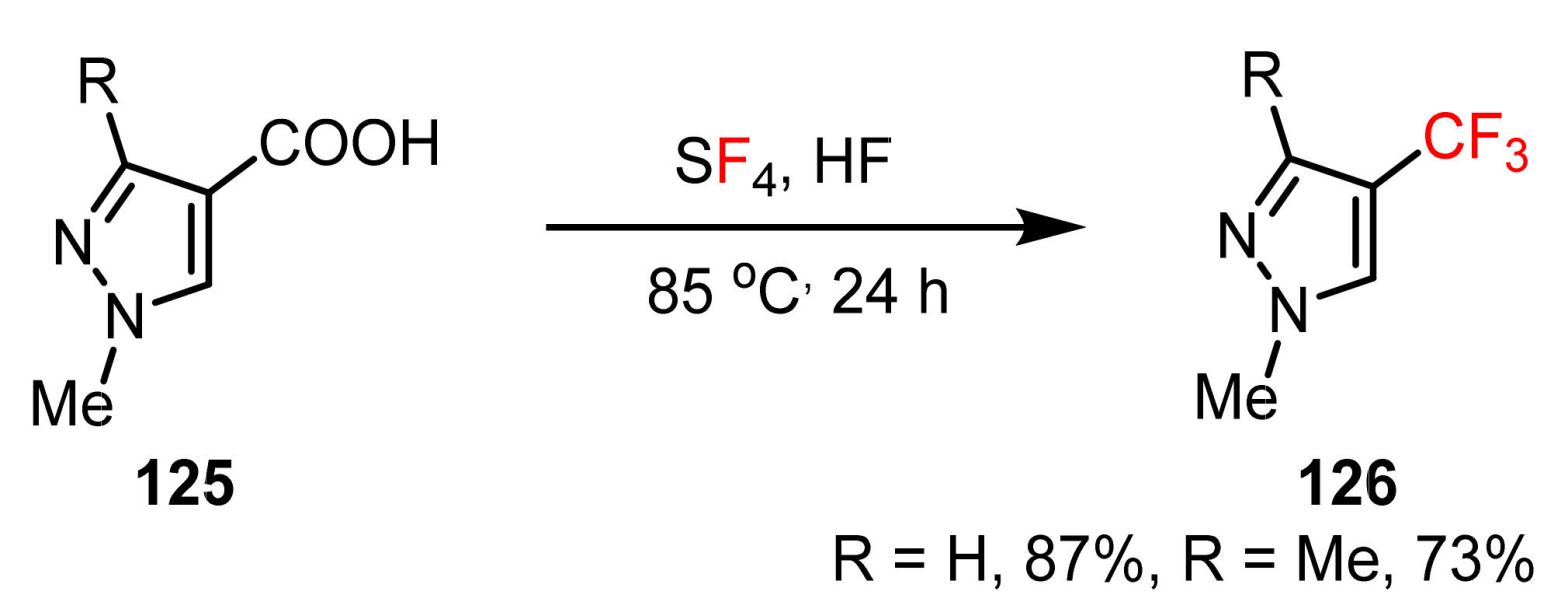
Direct fluorination to synthesize 4-trifluoromethylpyrazoles.

The above-mentioned methodologies offer great convenience in constructing trifluoromethylpyrazoles with various trifluoromethyl-containing building blocks and reagents. However, some of these trifluoromethyl-containing chemicals are expensive or potentially explosive [47,49]. Thus more straightforward methodologies like the direct fluorination of existing functional group into a trifluoromethyl have also been explored. For example, in the presence of HF, sulfur tetrafluoride can directly deoxofluoridize 4-pyrazolecarboxylic acids into corresponding 4-(trifluoromethyl)pyrazoles in high yields [50] (Scheme 42).

### 3.2. N-Alkylation, N-Arylation, and N-Alkenylation of Pyrazoles

**Figure 6:**
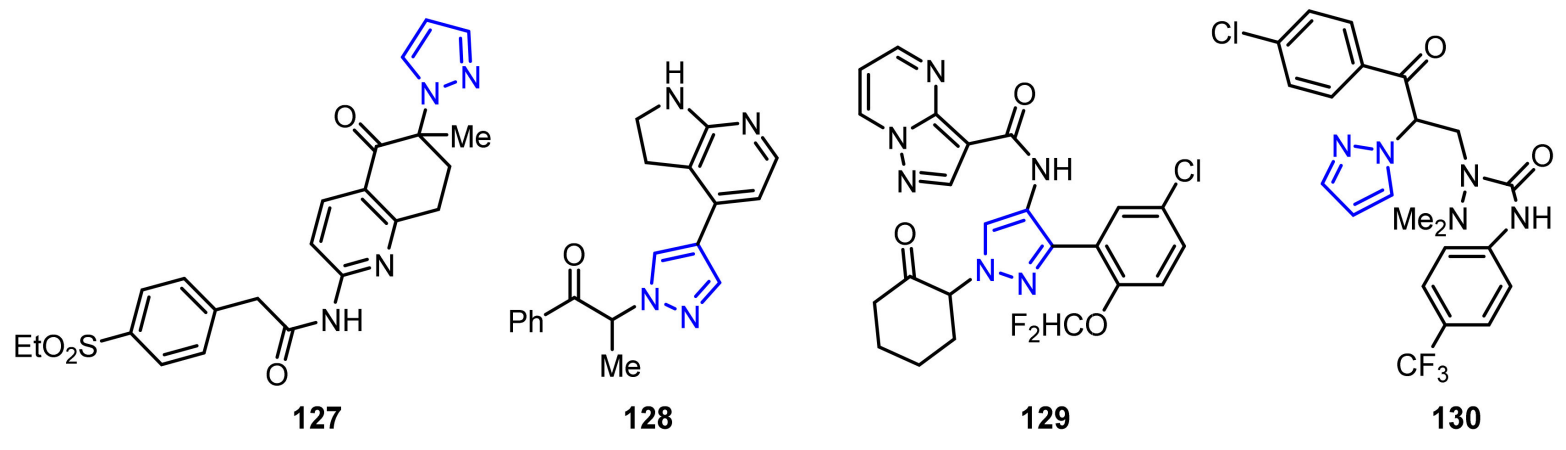
Applications of α-pyrazole ketone scaffolds in bioactive molecules.

The direct *N*-alkylation of pyrazoles to yield α-pyrazole ketone derivatives is of great importance because of their pharmacological potential in diverse bioactive molecules. Examples including retinoic acid receptor-related orphan receptor gamma (RORγ) modulator 1 (**127**), Janus kinase (JAK) inhibitors (**128**) and (**129**), and insecticides (**130**) are shown in Figure 6 [51]. High regioselectivity in such transformations is readily achieved under optimized reaction conditions. In addition, any minor impurity of regioisomer can be removed easily due to the differences in its physical properties (e.g., polarity, solubility difference) to the main product during purification.

#### 3.2.1. The Synthesis of *N*-Alkylated Pyrazoles

**Scheme 43:**
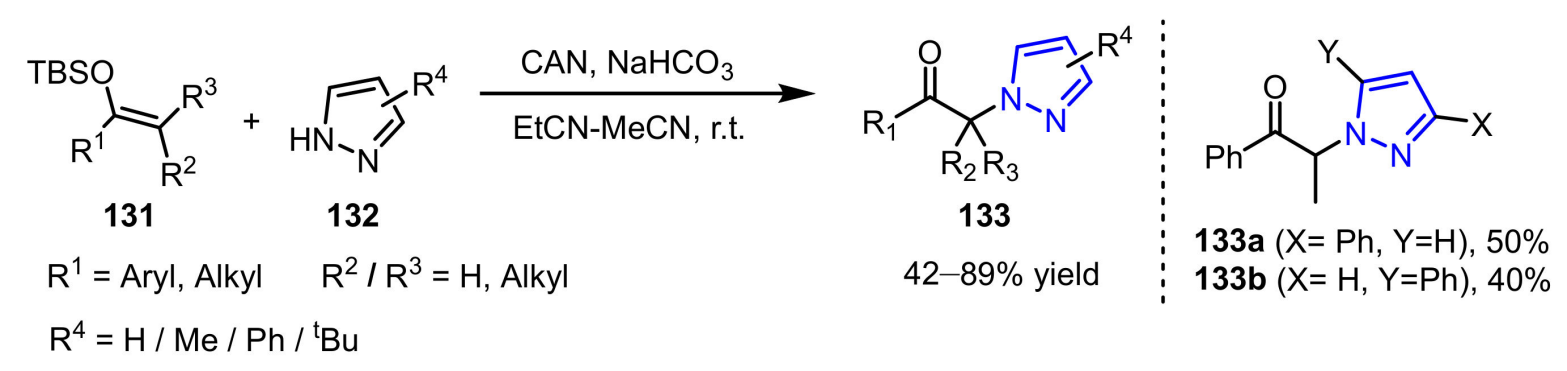
*N*-alkylation of pyrazoles.

Dhanju et al. reported a general method of using ceric ammonium nitrate to mediate oxidative coupling of enolsilanes and heterocycles for making diverse α-pyrazole ketones [51]. Interestingly, they found that under the same reaction conditions, quaternary α-pyrazole ketone derivatives could be prepared; such pyrazole derivatives are not generally accessible via conventional synthetic methods due to steric hindrance (Scheme 43). Nevertheless, for some asymmetrical pyrazole substrates, this method generated a mixture of regioisomers. For example, **133a** and **133b** were synthesized in a ratio of 1.25:1. This is attributed to the conjugation effect of the phenyl group at the C_2_ substitution of the pyrazole ring, the hyperconjugation inside the bicyclic ring system consisting of the pyraozle group, and the phenyl group accelerates the fast shifting of the C–N double bond between the two nitrogen atoms. The two flexible tautomeric structures tend to afford the product as a mixture of regioisomers in almost equal ratio. Conversely, other substitutions (i.e., H, *t*Bu) having no conjugation effect only provided a single regioisomer in high yield [51].

#### 3.2.2. The Synthesis of *N*-Alkenylated Pyrazoles

**Scheme 44:**
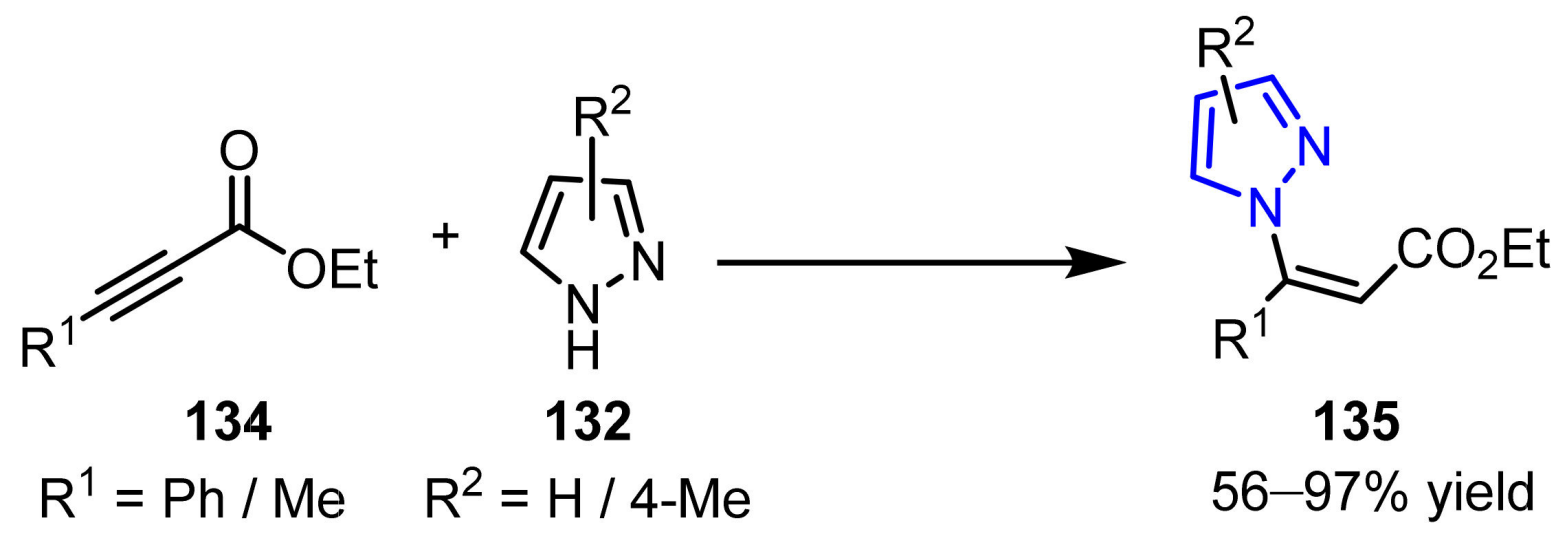
*N*-alkenylation of pyrazoles.

Compared to the *N*-alkylation and arylation of pyrazoles, few synthetic methods for *N*-alkenylation of pyrazoles were reported. In 2015, to prepare β-pyrazolyl acids possessing a wide range of bioactivities [52], Luo and co-workers [53] reported a gold-catalyzed stereoselective 1,4-conjugate addition of pyrazoles to propiolates to afford *N*-alkenyl substituted pyrazoles in moderate to good yields with high regioselectivity. Under optimized conditions, the undesired regioisomer ratio was <3% when using an unsubstituted pyrazole as the substrate. After hydrogenation of the double bond, β-pyrazolyl acid esters, the precursors of β-amino acids, could be obtained effortlessly (Scheme 44).

#### 3.2.3. The Synthesis of *N*-Arylated Pyrazoles

**Scheme 45:**
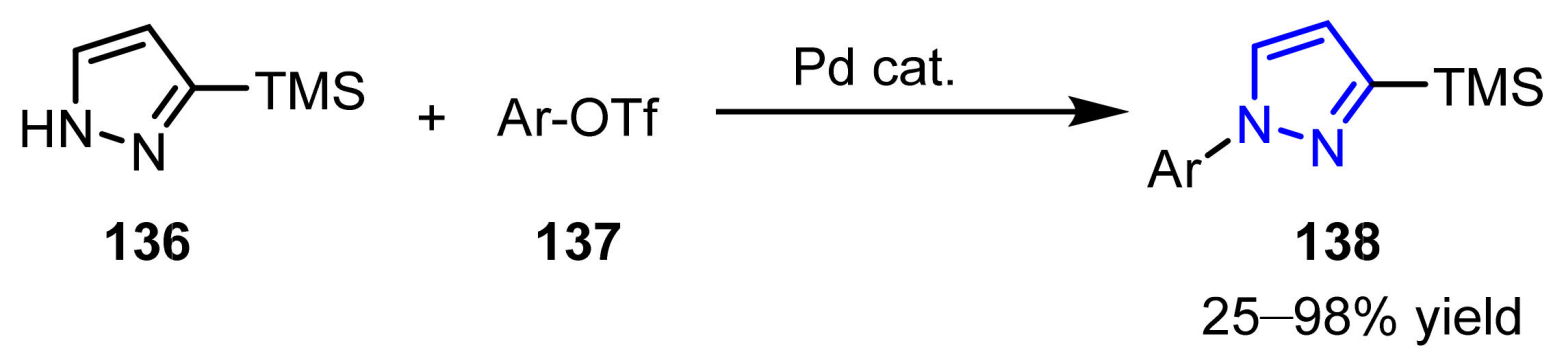
Pd-catalyzed synthesis of *N*-arylated pyrazoles.

*N*-arylpyrazoles motifs are common in natural products, bioactive molecules, and pharmaceuticals [54]; furthermore, they are also prevalent components of auxiliary ligands for transition-metal catalysis [55]. Conventional methods of synthesizing *N*-arylpyrazoles rely on using either Buchwald–Hartwig coupling [56] or classical Ullmann reactions [57], requiring noble palladium catalysts or harsh reaction conditions. Onodera et al. reported an efficient method using palladium-catalyzed coupling of aryl halides with 3-trimethylsilylpyrazole to make *N*-arylpyrazoles in quantitative yields [58]. Interestingly, 3-trimethylsilyl substitution on pyrazole substrates is essential for high yields; in addition, 3-trimethylsilyl substitution also offers considerable synthetic potential and diversity for subsequent structural modifications (Scheme 45).

**Scheme 46:**
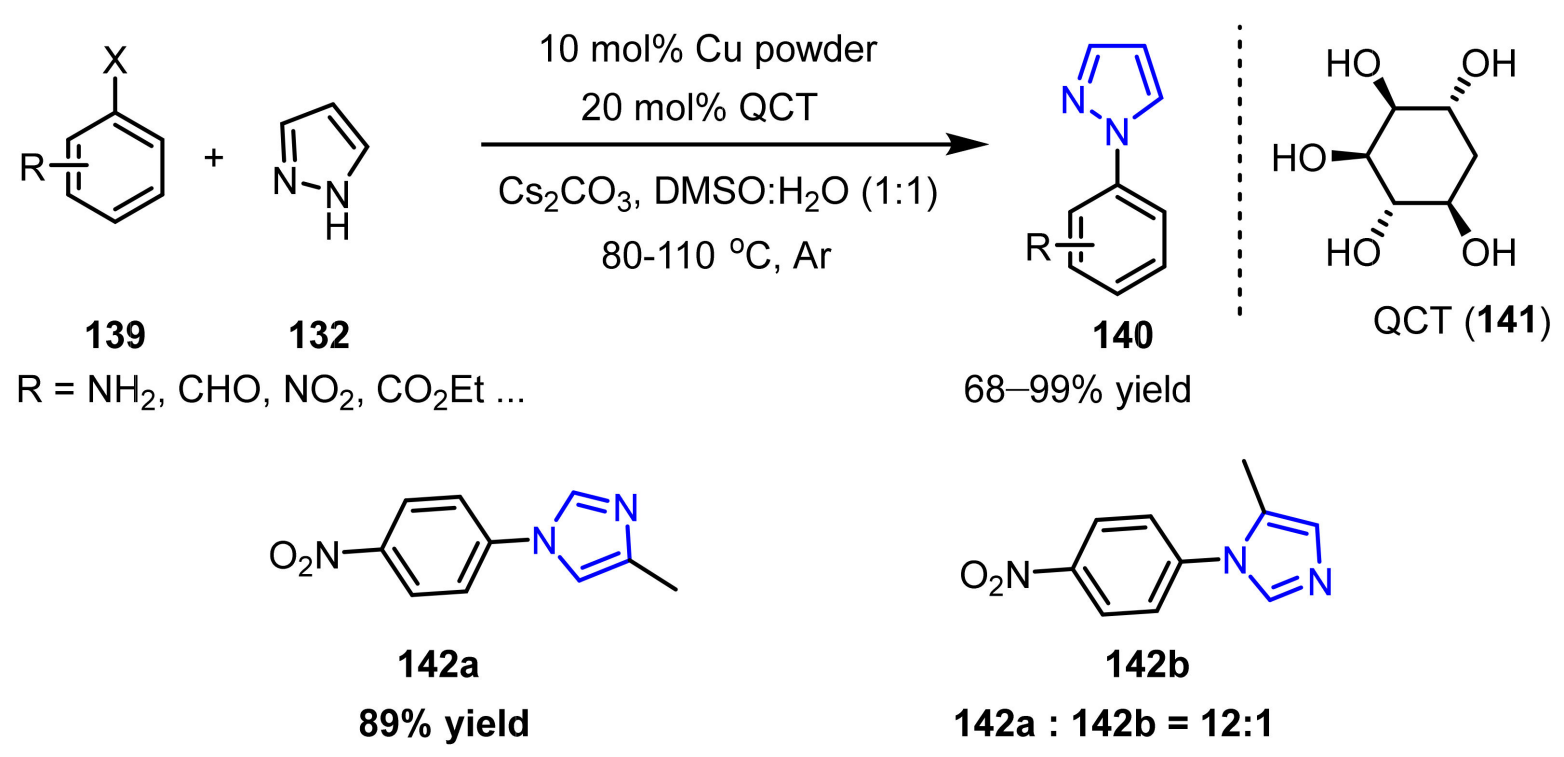
Cu-catalyzed synthesis of *N*-arylated pyrazoles.

A general Cu-catalyzed Ullmann-type coupling to synthesize *N*-arylazoles was reported by Zhou and co-workers [59]; they discovered that using L-(-)-quebrachitol (QCT), a recycled waste in natural rubber industry as the *O, O*-bidentate ligand, both aryl bromides and aryl chlorides can afford *N*-arylpyrazoles. In addition, aryl triflates that are readily made from phenolics were also well tolerated to prepare desired products in excellent yields (Scheme 46). Noticeably, using asymmetric imidazole having a tautomerism similar to pyrazole as the substrate, **142a** could be made in a high regioselective ratio of 12:1. This Cu powder involved catalytic system presented a superior regioselectivity and high yield compared to the Cu_2_O catalytic system [60].Copper powder is more reactive in the Ullmann-type reaction than Cu_2_O as a catalyst. In addition, the ligand (QCT) can coordinate the in situ formed copper ions to stabilize the reaction intermediates, a more stable tautomeric structure. The collective roles have a net effect of a higher regioselectivity [59].

**Scheme 47:**
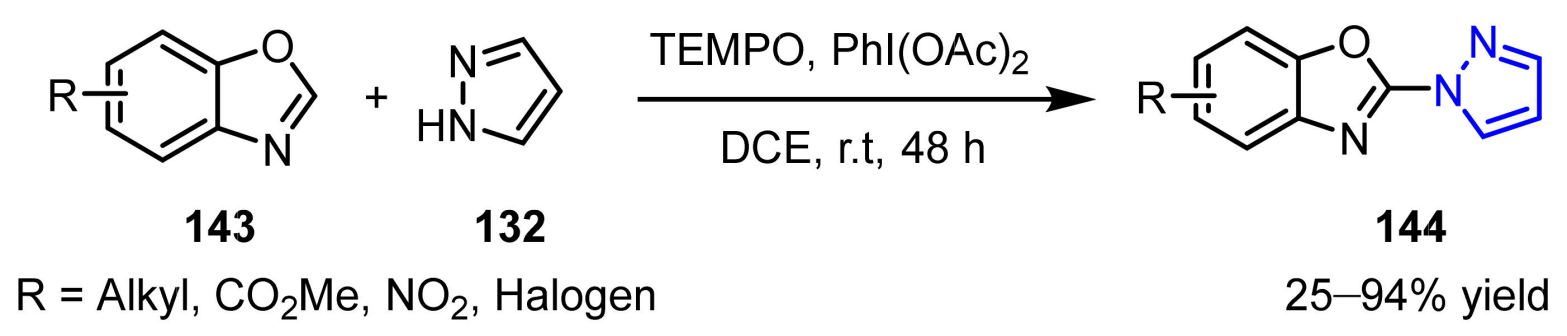
Oxidative coupling for the synthesis of *N*-arylated pyrazoles.

Wang et al. reported a metal-free oxidative methodology for the coupling of benzoxazoles and pyrazoles to form a bis-heterocyclic system of *N_1_*-benzoxazole substituted pyrazole derivatives [61]; the formation of radical species in the presence of oxidants is the common feature of such protocols (Scheme 47).

### 3.3. The Amination of Pyrazoles

**Figure 7:**
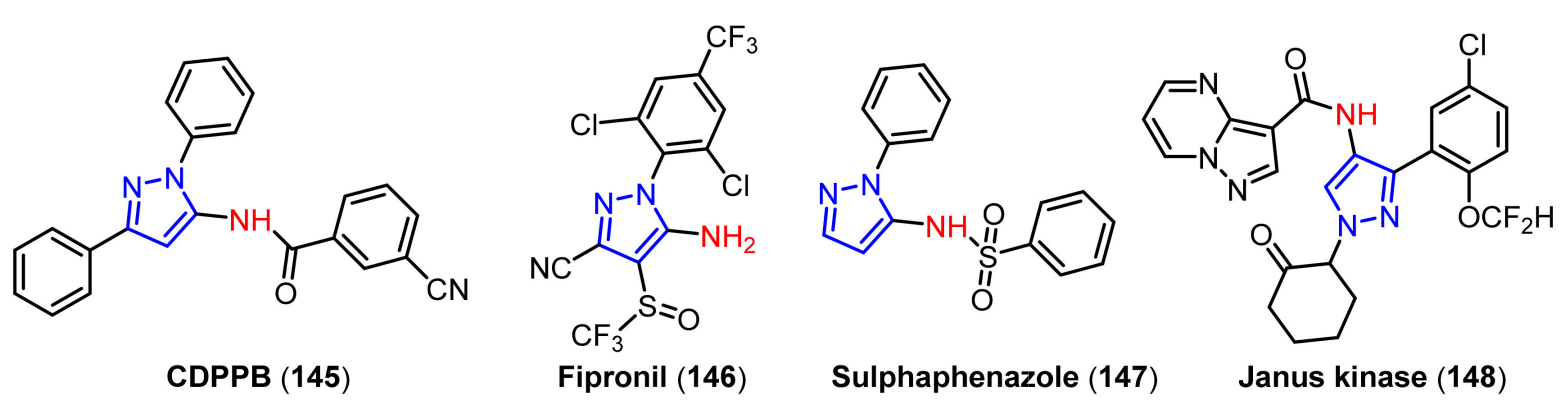
Bioactive aminopyrazoles.

Aminopyrazoles have broad applications in both pharmaceuticals and agrichemicals, examples such as CDPPB (**145**, positive allosteric modulators), fipronil (**146**, insecticide), sulfaphenazole (**147**, sulfonamide antibacterial), and a Janus kinase inhibitor (**148**) all bear an amino group at the 5-position (Figure 7). In addition, the introduction of an amino group onto pyrazoles is also synthetically beneficial for further transformation [62].

**Scheme 48:**
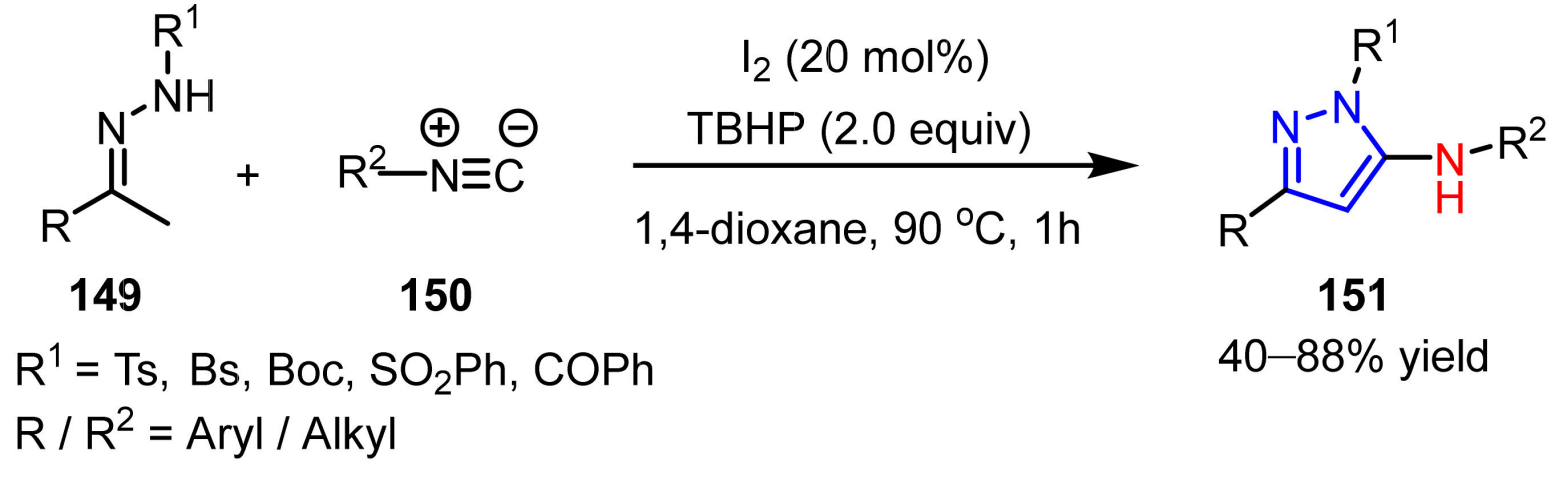
I_2_ catalyzed synthesis of 5-aminopyrazoles.

Senadi et al. reported an oxidative cross coupling of *N*-sulfonyl hydrazones with isocyanides through a formal [4 + 1] annulation process; this metal-free procedure generated 5-aminopyrazoles in good yields with broad substrate scopes [63] (Scheme 48).

**Scheme 49:**
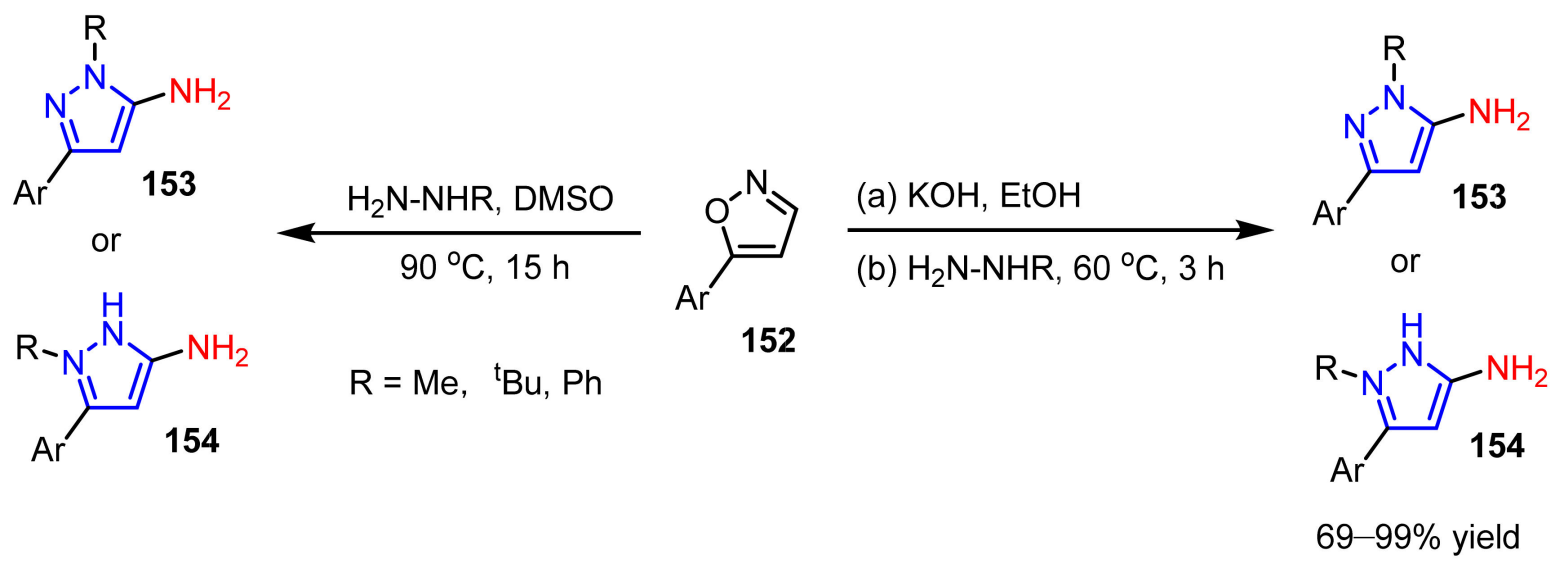
Isoxazoles as substrates for 3-aminopyrazoles synthesis.

Kallman et al. reported an efficient method to ammonolyze isoxazoles into aminopyrazoles either in DMSO using one-step, or in an ethanol solution of KOH using two consecutive steps [64]. Both procedures performed well to afford 3-aminopyraozles selectively. In situ NMR analysis indicated that this reaction underwent a ring-opening process of converting isoxazole into a ketonitrile intermediate, followed by the formation of (*Z*)-hydrazinylacrylonitrile, then a ring-closure step occurred to afford the target aminopyrazoles (Scheme 49). Unlike other methods providing high regioselectivity, the hydrolyzation of isoxazoles into aminopyrazoles involves the conversion of continuous intermediates. For example, the unstable intermediate hemiaminal can convert into either (*Z*)-hydrazone or (*E*)-hydrazone randomly, and this cis-trans isomerism was verified by in situ NMR analysis. The lack of a preponderant *Z* or *E* configuration leads to less regioselectivity [64].

### 3.4. The Selanylation of Pyrazoles.

**Scheme 50:**
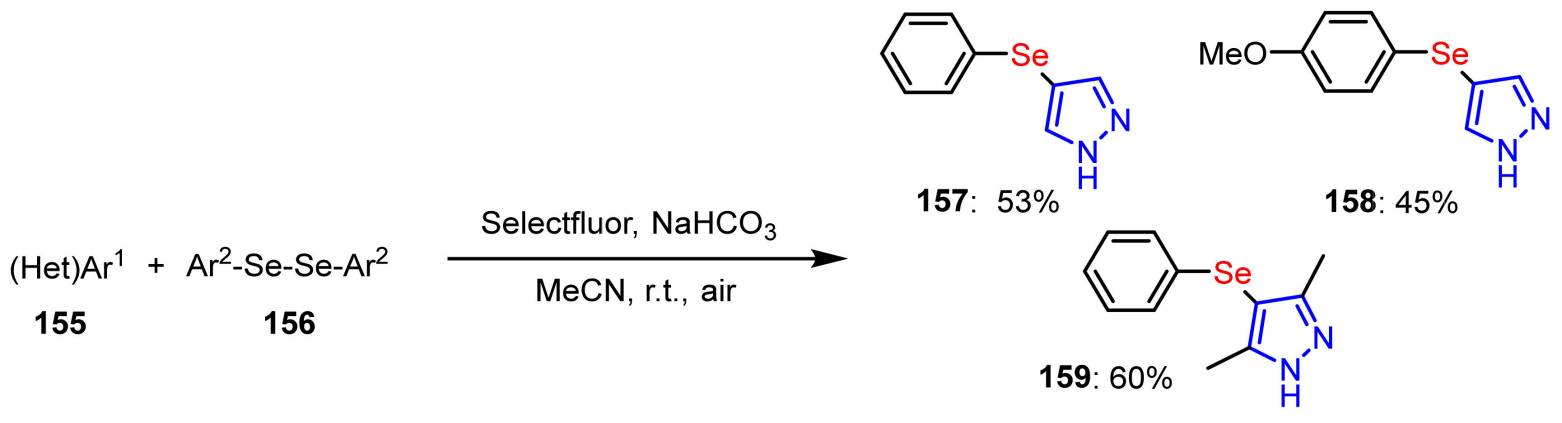
The synthesis of selanylated pyrazoles.

Emerging data from pharmacological studies and clinical trials reveal that selenoproteins and organoselenium molecules are participating in a variety of physiological activities [65,66]. For example, a selenium-containing celecoxib derivative was shown to be effective in downregulating the transcription of COX-2 and other pro-inflammatory genes [67]. This highlighted the significance of incorporating selenium into pyrazole motifs. Belladona et al. reported a direct C-H selanylation of pyrazoles with Selectfluor (*N*-chloromethyl-*N*-fluorotriethylenediammonium bis(tetrafluoroborate)), a stable and commercially available oxidant, to afford 4-phenylselenol substituted pyrazole derivatives [68]. The reaction was triggered by the interaction of diphenyl selenide with Selectfluor to form electrophilic selanyl species, which were then attacked by nucleophilic pyrazole substrates to give the final product. Mechanism studies suggest that the formation of a Se–F bond between Selectfluor and diselenide is the critical step to restrain the formation of non-fluorinated byproducts (Scheme 50).

### 3.5. The Borylation of Pyrazoles

**Scheme 51:**
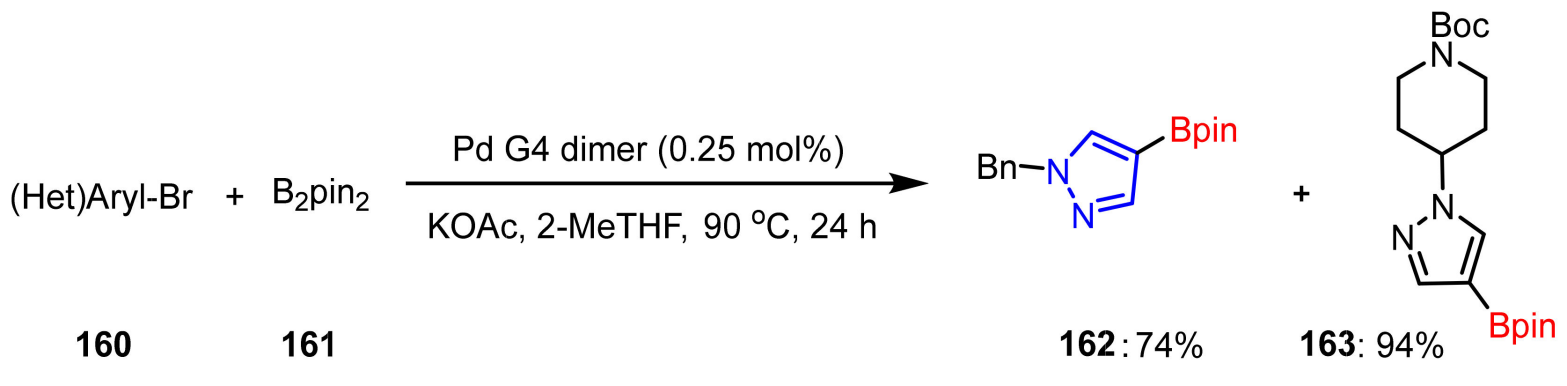
The synthesis of borylated pyrazoles.

Borylated pyrazoles are critical building blocks in scalable Suzuki-coupling reactions for preparing pyrazole-containing pharmaceuticals and fine chemicals. Nevertheless, such starting chemicals are commercially scarce due to the unavailability of relevant synthetic methodologies. Recently, Wang and co-workers [69] described that dianthphos, an easily prepared monophosphine ligand, is capable of facilitating the Miyaura borylation of pyrazole substrates with high yields, but only two representatives were presented in their publication (Scheme 51).

Above, we discussed the synthesis and functionalization of pyrazoles for different purposes. In the next section, we will focus on the implementation of pyrazoles as therapeutic drug candidates, particularly for treating neurological diseases such as Alzheimer’s disease (AD) and Parkinson’s disease (PD).

## 4. Applications of Pyrazoles in Neurodegenerative Diseases

Neurodegenerative diseases are characterized by the progressive loss of structure, function, and quantity of neurons, which result in clinical syndromes including motor, cognitive, and behavioral decline. Currently, Alzheimer’s disease (AD), and Parkinson’s disease (PD) are reported as the top two neurodegenerative diseases, and both are associated with abnormal protein aggregations within the brain. Although academic institutions and pharmaceutical industries have dedicated tremendous efforts to developing drug candidates for treating these two diseases, unfortunately, an effective therapeutic has not been identified yet. Patients and their families continue to bear a huge economic burden as well as enduring physical and emotional pressure. Meanwhile, diverse small-molecule drug candidates have been explored as potential therapeutics for AD and PD. In the development of potential drug candidates for the treatment of PD and AD, pyrazole scaffolds have two intrinsic advantages. First, the pyrazole heterocycle is a bidentate ligand: it can act as both a hydrogen-bond donor and a hydrogen-bond acceptor at the same time, thus allowing it to interact with carbonyl oxygen and amide proton on the peptide backbone complementarily [70]. Second, π–π-stacking interaction between pyrazole and phenylalanine in β-sheet peptides amplifies their high affinity toward peptide backbones [71]. Here, we will present an overview of recently reported potent pyrazole-containing biomolecules that have been evaluated for the treatment of AD and PD. As readers are encouraged to read the recent review by Gomes, Silva, and Silva [6], which summarizes the structures of pyrazole-containing diagnostic radiotracers for neurodegenerative disease, these are not discussed herein.

### 4.1. Applications of Pyrazoles in Alzheimer’s Disease (AD) Treatment

Although a precise understanding of the pathogenesis of AD remains elusive, several iconic hallmarks have been identified. For example, MRI studies on AD patients discovered significant hippocampal and entorhinal cortex atrophy compared to healthy age-matched control subjects [72]. Furthermore, histopathological examination of AD cases has shown that advanced disease is characterized by the deposition of senile plaques (SPs) and neurofibrillary tangles (NFTs), which are composed of aggregates of amyloid-β (Aβ) and hyperphosphorylated tau (ptau), respectively [73]. Based on anatomical studies, several hypotheses including (i) amyloid cascade hypothesis, (ii) tau hypothesis, (iii) metal ion dyshomeostasis, (iv) oxidative stress hypothesis, and (v) cholinergic hypothesis have been proposed in an attempt to guide drug discovery for AD treatment [74].

With respect to their corresponding protein targets, the leading pyrazole-containing therapeutic compounds can be divided into five classes: (1) acetylcholinesterase (AChE) inhibitors; (2) protein aggregation inhibitors; (3) PDE inhibitors; (4) dual leucine zipper kinase inhibitors; and (5) monoamine oxidase B inhibitors.

#### 4.1.1. Acetylcholinesterase (AChE) Inhibitors

Acetylcholine (ACh) is a critical neurotransmitter and neuromodulator in the central nervous system (CNS), its relatively low concentration in the cerebral cortex of AD brains results in the degeneration of cholinergic neurons as well as the deficit of cholinergic neurotransmission [75]. In neurons, ACh is synthesized from choline and acetyl-CoA when catalyzed by choline acetyltransferase (ChAT) and is hydrolyzed into acetate and choline by acetylcholinesterase (AChE) [76]. AChE is primarily found at the synaptic cleft and the neuromuscular junction of the brain. In contrast, outside of the CNS, the cholinergic enzyme subtype butyrylcholinesterase (BuChE) mainly exists in blood plasma. Unlike the substrate specific AChE, BuChE can nonspecifically hydrolyze many different choline or non-choline based esters and amides [77].The colocalization of AChE or BuChE with Aβ aggregates in SPs could accelerate Aβ aggregation, thus increasing neurotoxicity [78,79,80]. Therefore, cholinesterase inhibitors have been viewed as a promising target for AD treatment.

**Figure 8:**
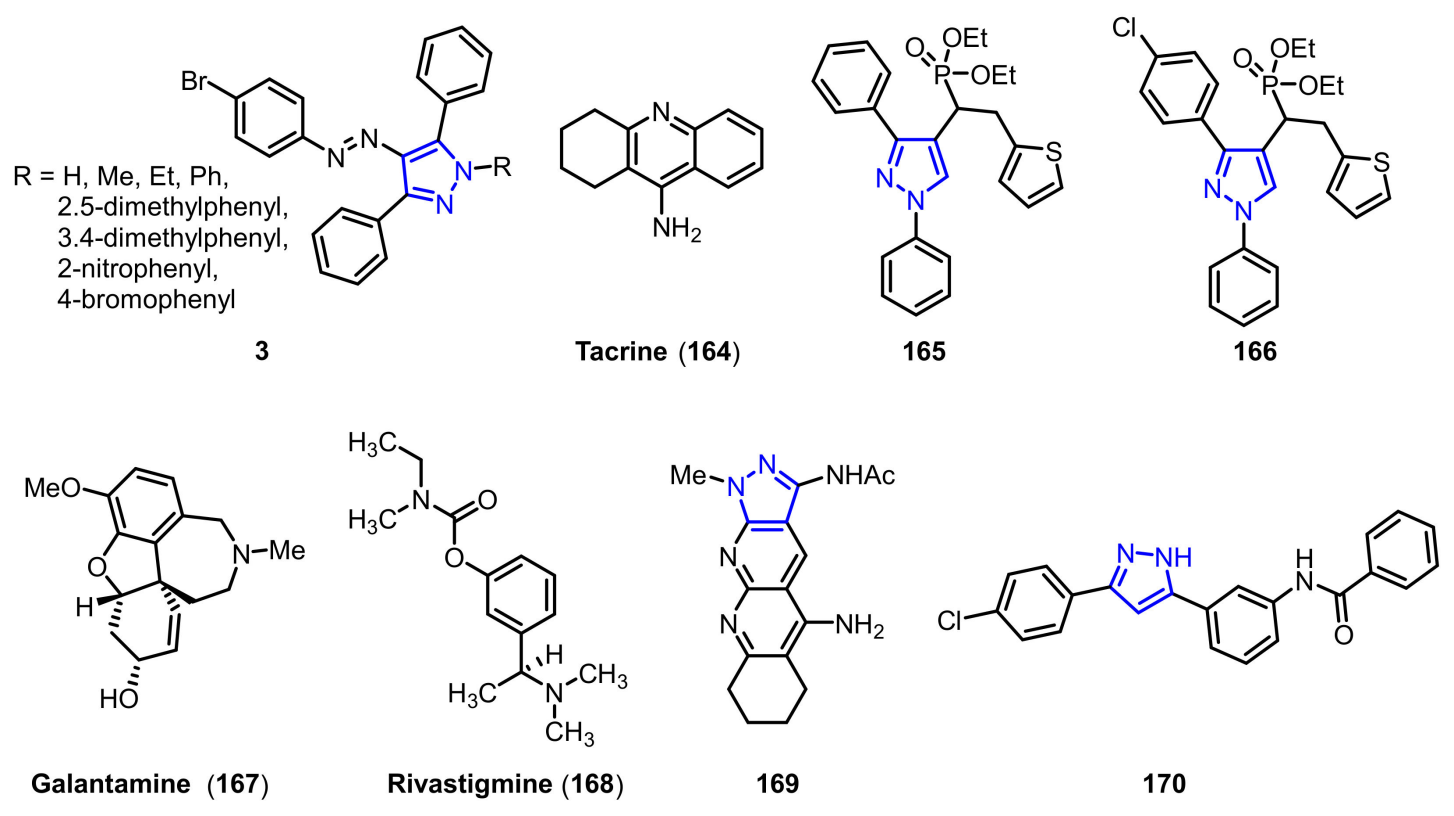
Chemical structures of cholinesterase inhibitors.

In 2018, Turkan et al. reported a series of substituted pyrazol-4-yl-diazene derivatives (**3**), and their in vitro enzymatic assay suggested that all were effective AChE and BuChE inhibitors with *K_i_* values at the nanomole level, better than tacrine (**164**), a discontinued acetylcholinesterase inhibitor that was used to treat AD [7]. However, their poor selectivity between AChE and BuChE, and broad activities over α-glycosidase and cytosolic carbonic anhydrase might be problematic. Shaikh et al. reported novel scaffolds of *N*-substituted pyrazole-derived α-aminophosphonates, two of these compounds (**165**, **166**) exhibited strong potency against AChE and high selectivity over BuChE, and their performances were better than the commercially available drugs galantamine (**167**) and rivastigmine (**168**) [81]. Compound **169** had ~100-fold selectivity for AChE over BuChE, furthermore, it exhibited 45% neuroprotection ratio in rotenone/oligomycin A-induced neuronal death. When compared to tacrine, only a minor activity decrease was observed [82]. Gutti et al. reported a linear pyrazole derivative (**170**) as an AChE inhibitor in 2019 [13]. When tested in MC65 cells at 50 μM, compound **170** improved cell viability by 90% and could reduce Aβ_1–42_ aggregation induced by metals effectively at 20 μM (Figure 8, Table 1).

#### 4.1.2. Protein Aggregation Inhibitors

The dysfunction and aggregation of Aβ and tau proteins are the main features of AD pathology. Aβ is a proteolytic product of amyloid precursor protein (APP), which is cleaved by α-secretase and γ-secretase to Aβ monomers with 38 to 43 amino acid residues, and studies have identified Aβ_42_ as a key monomer in pathology.[83,84]. These short Aβ monomers possess a propensity to aggregate through mutual recognition to form Aβ oligomers, which further aggregate into protofibrils and fibrils with a β-sheet conformation [85]. Aβ has also shown a seeding effect in the brain to transmit and expand its influence [86].

Tau is a phosphoprotein, its hyperphosphorylation can destabilize microtubules, thus causing invariably compromised axonal transportation, synaptic dysfunction, and signal transmission failure [87]. Furthermore, hyperphosphorylated tau tends to congregate into paired helical filaments (PHFs) and straight filaments (SFs) in steps [88]. In experimental mouse models of tauopathy, the accumulation of aggregated tau can disrupt anterograde axonal transport and impair long-term potentiation [89,90].

**Figure 9:**
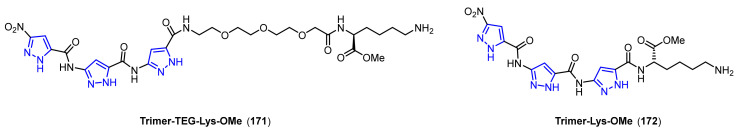
Chemical structures of pyrazole-containing Aβ_42_ inhibitors.

In 2011, Hochdörffer et al. developed a series of Aβ_42_ inhibitors based on a trimeric aminopyrazole carboxylic acid framework. They could convert well-ordered fibrils into less structured aggregates and thin bent filaments through backbone recognition and hydrophobic interactions with Aβ_42_ (Figure 9) [91]. Compound (Trimer-TEG-Lys-OMe, **171**) displayed the most potent inhibition and disaggregation activities against fibril formation, with ~80% of reduced thioflavin fluorescence absorption and 43% of increased PC-12 cell viability in an Aβ existing cytotoxic environment. Notably, the triethyleneglycol (TEG) spacer bridging trimeric aminopyrazole core and lysine appendix are essential to maintain the inhibition activity, as the spacer plays an active role in destabilizing the U-shaped turn of the Aβ protofilament. When the spacer was omitted (**172**), an accelerated aggregation was contrarily observed. Meanwhile, the attached extension (TEG-Lys-OMe, **171**) allows diversity; when it was replaced by a pentapeptide “LPFFD”, the corresponding new compound showed a substoichiometric IC_50_ value (3 µM). Considering that Aβ concentration is at nanomolar range in the brain, much lower than the concentration (10 µM) used in in vitro studies, a 3 µM IC_50_ value is remarkable.

**Figure 10:**
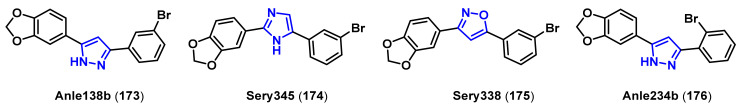
Chemical structures of pyrazole-containing inhibitors targeting tauopathy.

Anle138b (**173**) was first identified as a potent α-synuclein aggregation inhibitor when it was administered in vivo at nanomolar concentrations [92]. Later in vitro and in vivo studies carried in mouse models of AD suggested that it could also inhibit the formation of pathological tau aggregates by specifically binding to pre-aggregated tau species [93]. Molecular dynamic (MD) simulations revealed that anle138b (**173**) could block the conversion of ordered antiparallel β-strands into disordered β-sheet rich conformations by preferentially interacting with pre-aggregated tau fragments to reduce the overall number of intermolecular hydrogen bonds [94]. Structure-activity analysis showed that the pyrazole scaffold plays a crucial role in anle138b’s inhibitory effect; when the pyrazole moiety was replaced by imidazole (sery34*5*, **174**) or isoxazole (sery338, **175**), the anti-aggregation effect was weakened due to altered hydrogen bond characteristics. Interestingly, pyrazole anle234b (**176**), a structural isomer of anle138b, is biologically inactive. Anle234b (**176**) is more sterically hindered than anle138b due to placement of the bulky bromine group at the ortho- rather than meta-position, which creates a more stable/less flexible conformation. The torsional inactivation of anle234b (**176**) hampers the interactions of the bromophenyl and pyrazole ring with peptide backbones, resulting in a reduced hydrogen bond strength (Figure 10) [92,94].

**Figure 11:**
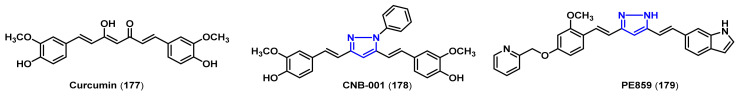
Chemical structures of pyrazole-containing curcumin derivatives.

Curcumin (**177**), a natural phenolic compound found in the rhizome of Curcuma longa, has been used to treat various ailments in India for centuries. Structural modifications of curcumin to improve its instability and bioavailability have been an attractive strategy in new drug discovery [95,96]; several pyrazole-containing curcumin derivatives have showed potency for AD treatment [97]. CNB-001 (**178**), a pyrazole derivative of curcumin, was initially found to have broad neuroprotective activity [98,99]. Subsequent studies in AD animal models revealed that it could also promote Aβ clearance and improve memory [100]. Kotani et al. further found that CNB-001 (**178**) could stimulate the expression of the endoplasmic reticulum chaperone glucose-regulated protein 78 (GRP78) and increase formation of the amyloid precursor protein APP/GRP78 complex. The subsequent downregulation of intracellular APP trafficking results in a reduced Aβ production without inhibiting β- or γ-secretase activity [101]. Okuda et al. developed the inhibitor PE859 (**179**), this curcumin derivative has IC_50_ values of 1.2 µM and 0.66 µM, respectively, to inhibit Aβ and tau aggregations, and improved in vivo pharmacokinetics and pharmacological efficacy, making it is more competitive as an AD drug candidate than curcumin [102]. Furthermore, studies with PE859 (**179**) in the senescence-accelerated mouse prone 8 (SAMP8) model of accelerated aging showed it also protected cells from Aβ-induced cytotoxicity damage by decreasing Aβ and tau aggregations in the mouse brains (Figure 11) [103].

#### 4.1.3. Phosphodiesterase (PDE) Inhibitors

Age-related cognitive decline and synaptic dysfunction are closely associated with decreased cAMP (cyclic guanosine monophosphate) and/or cGMP (cyclic guanosine monophosphate) concentration in the brains caused by increased phosphodiesterase (PDE) expression [104,105]. Therefore, PDE inhibitors that can specifically bind with high affinity to specific PDE isomers like PDE1, PDE5, or PDE9 to reduce the hydrolyzing of cAMP and/or cGMP have been evaluated for pharmaceutical potential in clinical trials for the treatment of AD [106].

**Figure 12:**
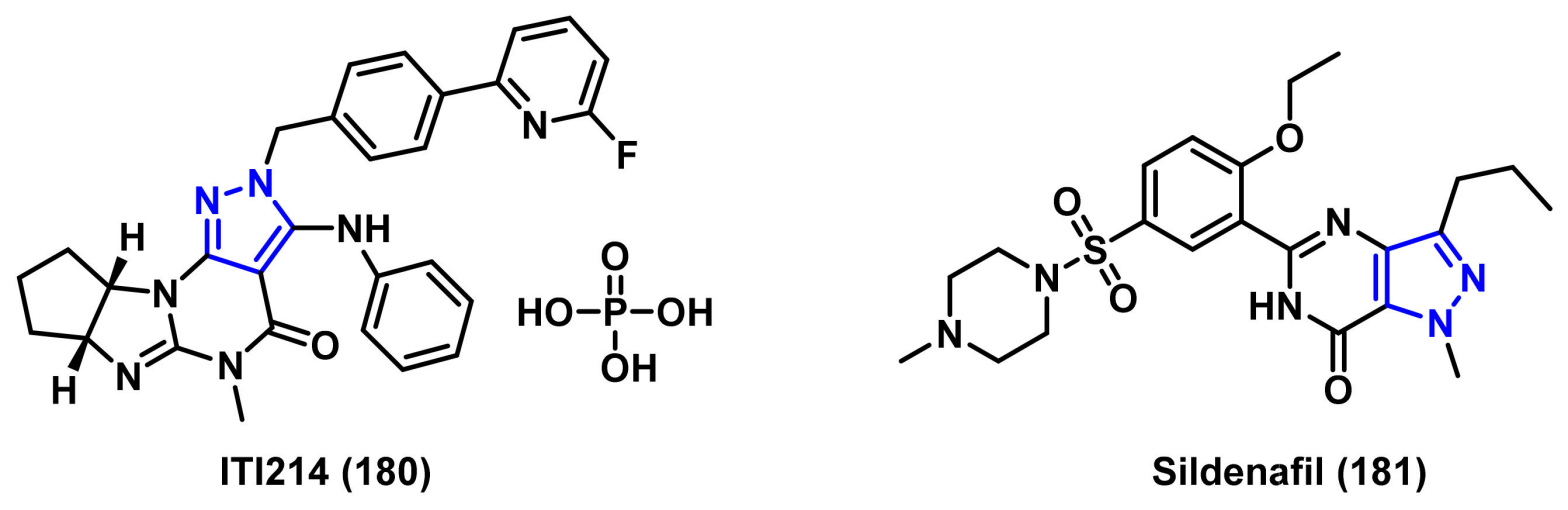
Chemical structures of pyrazole-containing PDE1 and PDE5 inhibitors.

The PDE1 group has three isoforms: PDE1A, 1B, and 1C, their activities are regulated by intracellular calcium and calmodulin (CaM) [104]. In human and rodent brains, PDE1A is predominantly expressed in the hippocampus, cortex, striatum, thalamus, and cerebellum; PDE1B is prevalent in the hippocampus, cortex, striatum; while PDE1C is more ubiquitously found in the cortex, cerebellum, and amygdala [107,108]. ITI214 (**180**), a potent and highly selective PDE1 inhibitor (*K_i_* = 0.058 nM), which is still in clinical trials, was developed by Intra-Cellular Therapies. This inhibitor was able to significantly enhance memory performance in vivo with a minimum effective dose of 3 mg/kg in various rat models (Figure 12) [109].

The prominent expression of PDE5 in smooth muscles has led to the extraordinary success of PDE5 inhibitors including sildenafil (**181**), vardenafil, and tadalafil for the treatment of erectile dysfunction [105]. Furthermore, PDE5 is also expressed in human hippocampus and frontal cortex, and particularly abundant in Purkinje neurons [110,111]. Studies in different AD mouse models have indicated that sildenafil (**181**) can improve synaptic function, restore memory loss, and upregulate CREB phosphorylation signaling to reduce Aβ levels over the long-term [112,113]. When administrated to anile Tg2576 transgenic mice (15 mg/kg, intraperitoneally), sildenafil (**181**) could completely reverse cognitive impairment and reduce tau hyperphosphorylation in the hippocampus [107]. More importantly, clinical trials of sildenafil (**181**) in healthy humans showed no central nervous system side effects. Although no overt effect on spatial auditory attention or visual word recognition was observed, sildenafil does help information processing and change specific components of event-related potentials with enhanced attention, and also reduced negativity of electroencephalogram in a memory task [114].

**Figure 13:**
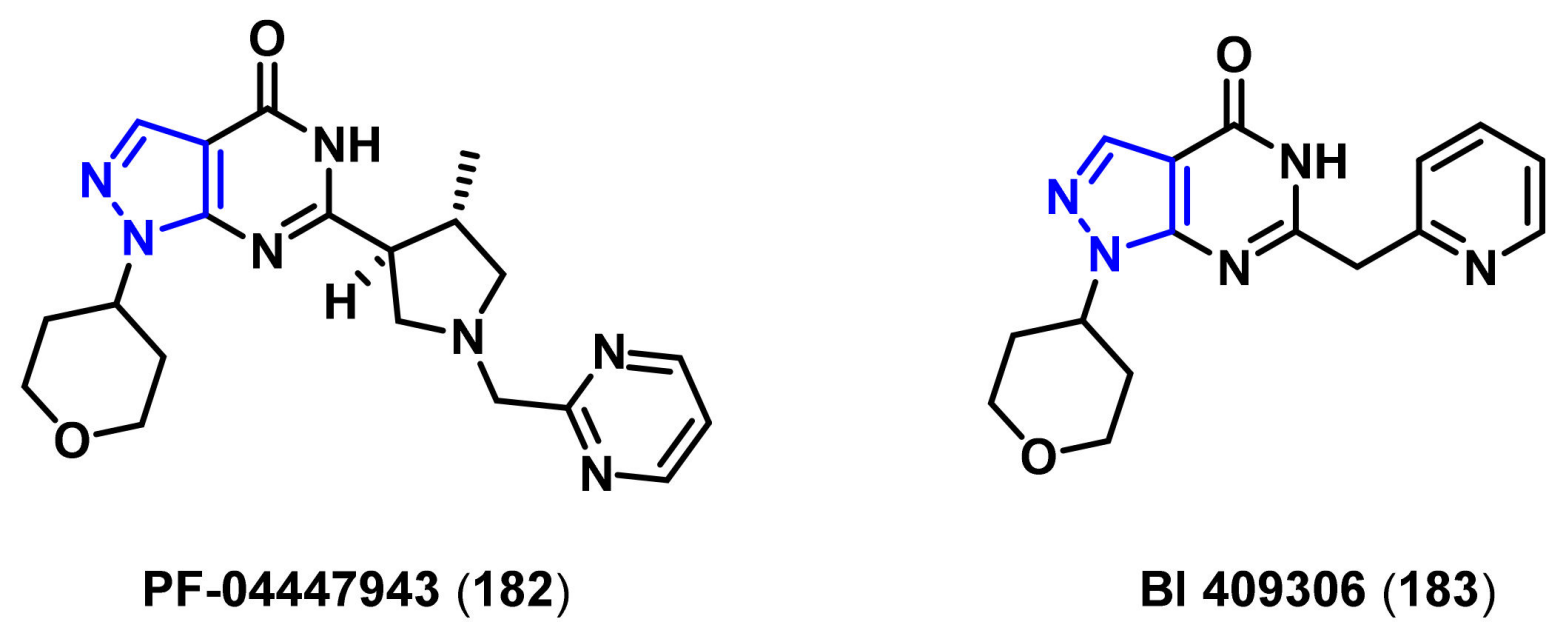
Chemical structures of pyrazole-containing PDE9 inhibitors.

PDE9A is primarily expressed in the brain, with high concentrations in the cerebellum, neocortex, striatum, and hippocampus [115]. Consistent with the specific function of cGMP in controlling neurotransmission and enhancing hippocampal synaptic plasticity, the inhibition of PDE9A results in significant accumulation of cGMP in cerebrospinal fluid of nonhuman primates and humans, thus damaging brain functions like sensory processing, learning, and memory [116]. Compound PF-04447943 (**182**) (*K_i_* = 8.3 nM), a PDE9A inhibitor developed by Pfizer, was found to increase cGMP concentration in cerebrospinal fluid by ~23-fold when dosed in rats, monkeys, and humans [117]. Unfortunately, no cognitive improvement was observed in subsequent phase II clinical trials when administered twice a day for 12 weeks to moderate stage AD patients. Instead, it resulted in a higher incidence of serious adverse events compared to the placebo group [118]. BI 409306 (**183)** is another potent PDE9A inhibitor, and has IC_50_ values of 65 nM and 168 nM in human and rat, respectively [119,120]. Preclinical studies demonstrated that BI 409306 (**183)** could increase cGMP concentrations in rodent prefrontal cortex and cerebrospinal fluid, and promoted long-term potentiation, and improved episodic and working memory performance [120]. Although it was well tolerated in prodromal to mild stage AD patients, its phase II clinical trial was discontinued because no visible efficacy in improving cognitive function was observed (Figure 13) [120].

#### 4.1.4. Dual Leucine Zipper Kinase (DLK) Inhibitors

**Figure 14:**
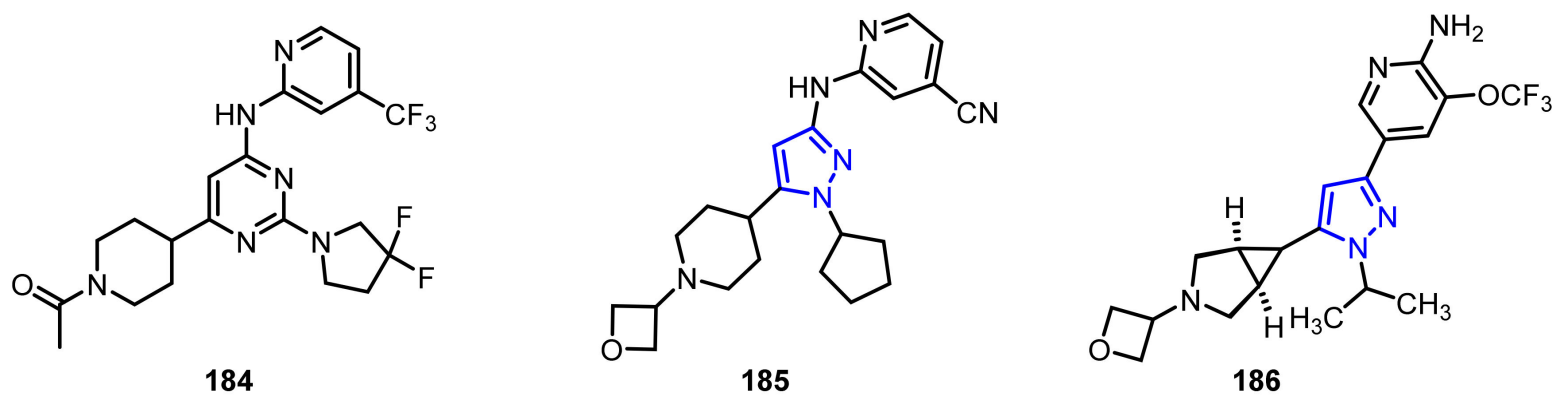
Chemical structures of pyrazole-containing dual leucine zipper kinase (DLK) inhibitors.

Dual leucine zipper kinase (DLK, MAP3K12) mediates axon degeneration and neuronal apoptosis after activation [121]. DLK-inducible knockout mice displayed increased synaptic transmission and reduced neuronal degeneration when faced with neuronal insult [122]. Ex vivo imaging found that Aβ/plaque-associated synaptic loss is at least partially mediated by DLK signaling [123]. Experiments in mouse models of AD demonstrated that DLK deletion can reverse cognitive deficits and rescue certain phenotypical behaviors. Although the elimination of DLK did not reduce Aβ_42_ production, plaque load, or alter tau pathology, DLK-knockout mice had higher levels of full-length amyloid precursor protein (APP), amyloid plaque, plaque-associated gliosis, and enhanced cell survival in the subiculum of tau [123]. In 2015, Patel et al. reported the first small molecule DLK inhibitor **184** that effectively reduced c-Jun phosphorylation in nerve crush and 1-methyl-4-phenyl-1,2,3,6-tetrahydropyridine (MPTP)-induced neuronal injury [124]. The inhibitor **185** (DLK *K_i_* = 42 nM) was developed using a scaffold-hopping strategy, in which the pyrimidine core was replaced by a pyrazole; it exhibits comparable pharmacological properties and inhibitory activities to compound **184** in a rat model [125]. After further optimization, a more potent compound **186** (DLK *K_i_* = 3 nM) was identified. It had lower plasma clearance (total and unbound), a moderate volume of distribution, a long biological half-life, and good bioavailability when dosed in cynomolgus monkeys. A single dose of **186** (50 mg·kg^−1^) resulted in significantly decreased phosphorylation of c-Jun, leading to a near-complete inhibition of JNK when tested in a mouse model of AD (Figure 14) [126].

#### 4.1.5. Monoamine Oxidases B (MAO-B) Inhibitors

Monoamine oxidases (MAOs) are mitochondrial outer membrane-bound enzymes, existing as two distinct enzymatic isoforms, MAO-A and MAO-B, and are responsible for the metabolism of neurotransmitters such as dopamine, serotonin, adrenaline, and noradrenaline [127]. MAO-B inhibitors selegiline and rasagiline have been used to treat Parkinson’s disease clinically. In addition, MAO-B inhibitors are also potential therapies for AD. A post-mortem brain study of AD cases indicated that MAO-B activity increased dramatically in three cortical regions (frontal, parietal, and occipital cortices), thalamus, and white matter [128], the observation was verified by another independent study [129]. MAO-B activity also increases in association with gliosis, resulting in elevated reactive oxygen species (ROS) levels, which in turn enhances Aβ production by decreasing the activity of α-secretase while enhancing the activity of β- and γ-secretases [130,131]. Studies suggest that MAO-B inhibitors like selegiline could slow AD progression in patients suffering from moderately severe impairment [132].

**Figure 15:**
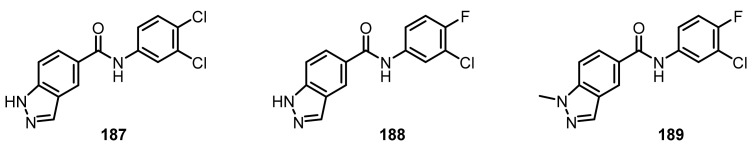
Chemical structures of monoamine oxidases (MAO) inhibitors.

A new strategy for tackling the complexity of AD pathology is incorporating several pharmacological scaffolds into a single molecular entity to produce a synergistic effect [133]. This tactic turned out to be useful when fusing pharmacological scaffolds from a MAO-B inhibitor and an AChE inhibitor together to afford multi-target-directed lead compounds as potential therapeutics against AD [134,135]. Tzvvetkov et al. reported three potent, reversible, and competitive MAO-B inhibitors (**187–189**) with high selectivity against the MAO-A isoform [136]. Enzyme studies suggest that all of them have the ability to bind to Fe(II) and Fe (III) via UV–Vis. These water-soluble, highly blood–brain barrier permeable MAO-B inhibitors are promising drug and radioligand candidates as diagnostic and therapeutic agents for AD (Figure 15, Table 2).

### 4.2. Applications of Pyrazoles in Parkinson’s Disease (PD) Treatment

Parkinson’s disease (PD) is the second most prevalent chronic neurodegenerative disease; it affects 1–2% of individuals over 60 years in the U.S. Initially, the most evident motor symptoms of PD are bradykinesia, muscle rigidity, and tremor; as the disease progresses, neurological symptoms including depression, pain, and sleep disturbance become more prevalent. Pyrazole derivatives have wide applications for PD treatment, and their roles can be divided into four classes: (1) antioxidants; (2) protein aggregation inhibitor; (3) adenosine A_2A_ receptor antagonists; and (4) PDE10A inhibitors.

#### 4.2.1. Antioxidants

**Figure 16:**
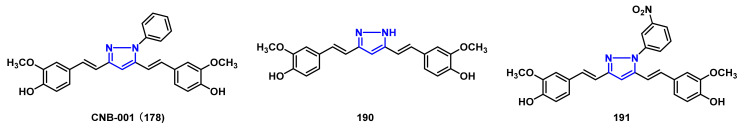
Chemical structures of pyrazole-containing antioxidants.

Mitochondrial dysfunction and subsequent oxidative stress are the main culprits leading to dopaminergic neuronal death in PD. Thus, antioxidants are potentially beneficial therapeutics in PD. Jayaraji and co-workers [137] reported that 2 µM of CNB-001 (**178**) could enhance cell viability insulted by reactive oxygen species (ROS). When applied to adult mice in a model of PD [137], CNB-001 (**178**) could significantly attenuate motor impairment, increase dopamine levels, and reduce inflammation. Two CNB-001 derivatives (**190**, **191**) [138] are also promising therapeutic inhibitors for the treatment of PD caused by α-synuclein amyloidosis, both of them have potent activity in preventing wide-type α-synuclein aggregation and fibrilization, with two-fold higher activity than their isoxazole analogues (Figure 16) [138].

#### 4.2.2. Protein Aggregation Inhibitors

**Figure 17:**
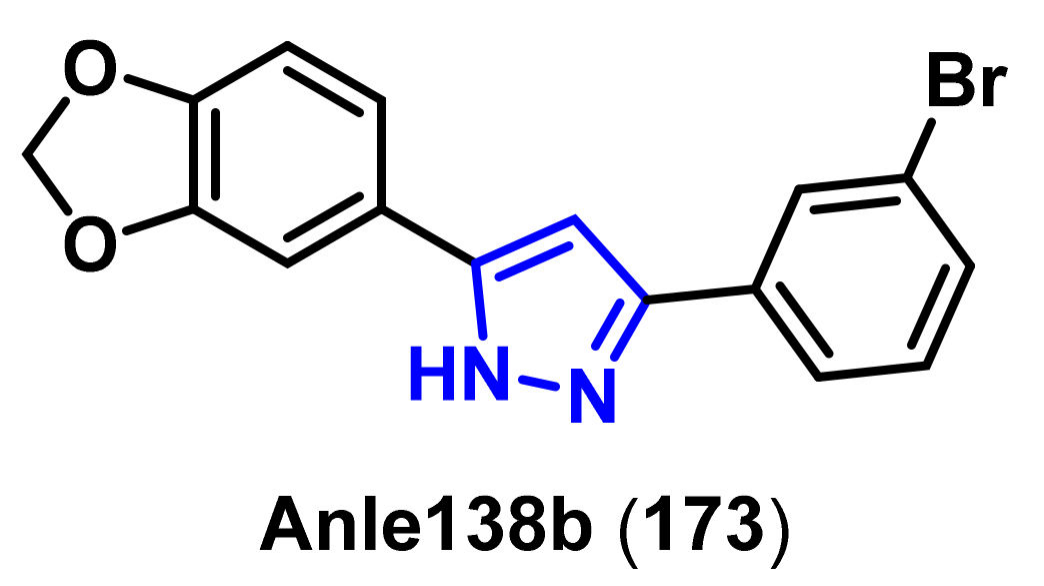
Chemical structure of a protein aggregation inhibitor.

Anle138b (**173**) was first identified as a pharmaceutical candidate in inhibiting the aggregations of prion protein and of α-synuclein [92]. In different mouse models of PD, anle138b (**173**) strongly inhibited α-synuclein accumulation and neuronal degeneration and had an excellent oral bioavailability, while no detectable toxicity at therapeutic doses was observed [92]. When co-incubated with α-synuclein fibrils, fluorescence spectrum indicated that it binds to the hydrophobic pockets of the fibril with good affinity (*K_d_* = 190 ±120 nM) (Figure 17) [139].

#### 4.2.3. Adenosine Receptor A_2A_ Receptor Antagonists

**Figure 18:**
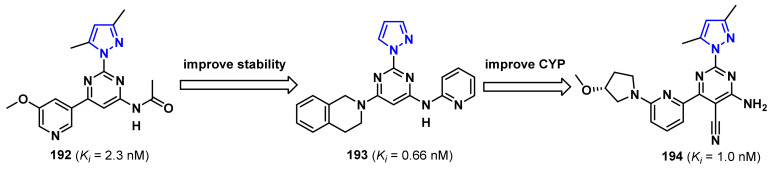
Chemical structures of pyrazole-containing A_2A_ receptor antagonists.

Adenosine receptor A_2A_ is abundantly expressed and co-localized with dopamine D_2_ in the striatum. The blockade of the A_2A_ receptor might antagonize dopaminergic neurotransmission in aspects relevant to motor control in PD patients [140], and the effect of A_2A_ antagonists has been supported by studies on rodent and primate PD models as well as preliminary clinical observations [141,142]. Pyrazole scaffolds have been widely employed in A_2A_ antagonists [143], for example, preladenant is discontinued due to lack of efficacy. Compound **192 [144]** exhibited exceptional receptor binding affinity and ligand efficiency against adenosine A_2A_ receptor (*K_i_* = 2.3 nM), however, the 4-acetamide on the pyrimidine ring is unstable and tends to hydrolyze in acidic environments. To overcome this issue, a structurally stable compound **193** (*K_i_* = 0.66 nM) was optimized, unfortunately, it was subject to moderate cytochrome P450 (CYP) inhibition (80% inhibition of CYP3A4 at 10 μM) [145]. Docking experiments based on crystal structure models of the adenosine receptor revealed that the 5-position of the pyrimidine ring was surrounded by multiple residues, which could serve as potential hydrogen bond donors or acceptors in the binding pocket. Analog **194**, which incorporated a cyano group, showed excellent potency and ligand efficiency with low cytochrome P450 inhibition [146]; when dosed orally as low as 3 mg/kg in rats, it could significantly reverse haloperidol-induced catalepsy (Figure 18).

#### 4.2.4. Phosphodiesterase-10A (PDE10A) Enzyme Inhibitors

Phosphodiesterase 10A (PDE10A) is a dual substrate phosphodiesterase enzyme that can hydrolyze both cAMP and cGMP [139,147]. The PDE10A/cAMP interaction is essential for dopamine neurotransmission, and has been implicated in the pathophysiology of Parkinson’s disease [148]. Investigators have reported that alteration of PDE10A expression is associated with progression and severity of patients with parkinsonism [149].

Tremendous effort has been dedicated to developing PDE10A inhibitors for the treatment of Parkinson’s disease and other neuropsychiatric disorders characterized by regulating medial striatal neuron (MSN) activity. Meanwhile, selectivity of an inhibitor for PDE10A over the other subtypes is also a critical issue. Numerous studies indicate that inhibition of PDE3A/B can lead to arrhythmia and increased mortality [150]; PDE4 plays an important role as a regulator of central nervous system function while inhibition of PDE4A can increase heart and respiratory rates [151].

Among the multiple PDE10A inhibitors, 2-[4-(1-methyl-4-pyridin-4-yl-1*H*-pyrazol-3-yl)-phenoxymethyl]-quinoline (MP-10) is highly potent (IC_50_ = 0.37 nM), selective (>100-fold), and has been extensively evaluated as a therapeutic inhibitor. While MP-10 has progressed to clinical trials without success [152], other PDE10A analogues are under evaluation, though there is no currently FDA-approved PDE10A inhibitor for treating PD.

**Figure 19:**
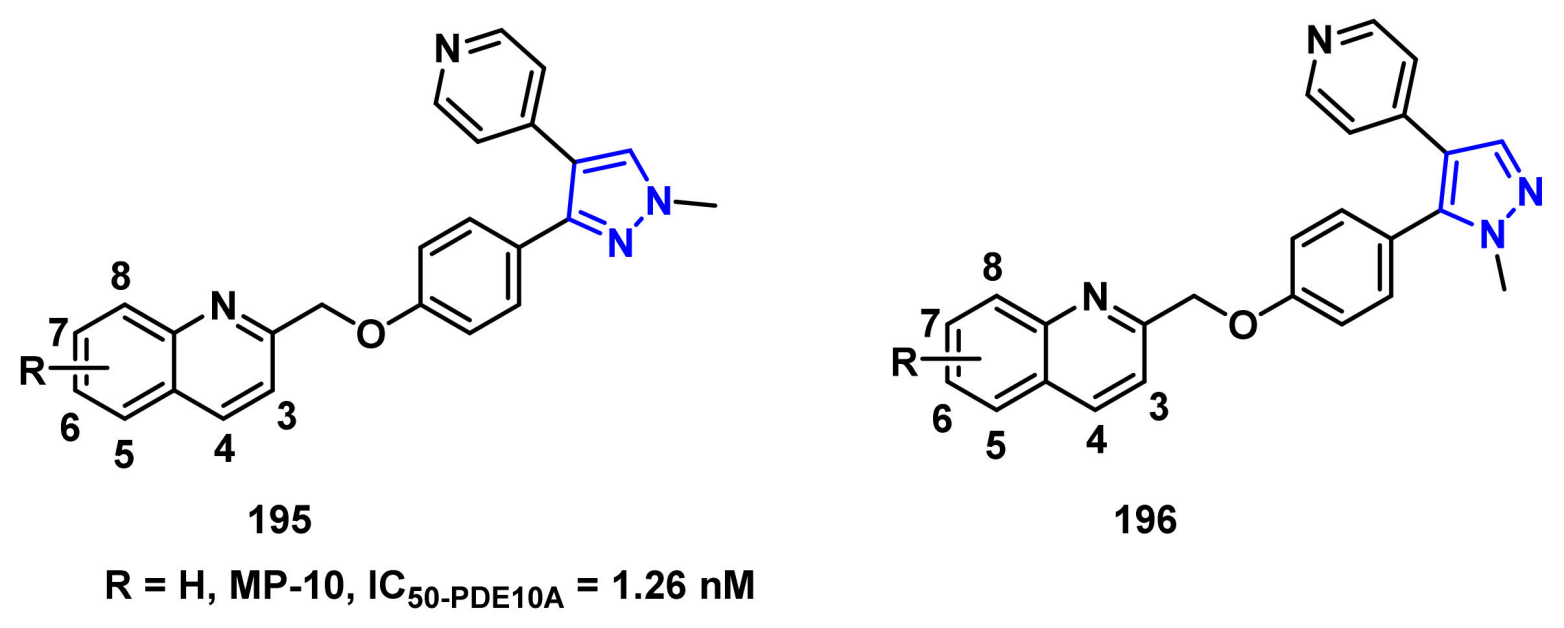
Chemical structures of pyrazole-containing PDE10A inhibitors.

Tu et al. reported a series of structurally related analogues of MP-10 in which a methoxy group (-OCH_3_) was added into the 3-, 4-, or 6-position of the 2-methylquinoline moiety of the MP-10 pharmacophore (Figure 19) [153]. The most potent molecule was the 3- and 4-methoxy substituted quinolines (**195)** with an O atom bridge linkage that showed potency similar to MP-10. Interestingly, little difference was observed in the in vitro biological activity of regioisomer **196** (Table 3). More importantly, the six compounds **195a–c** and **196a–c** not only had high potency for PDE10A, but also high selectivity for PDE10A versus PDE3A/3B and 4A/4B (Table 3).

**Figure 20:**
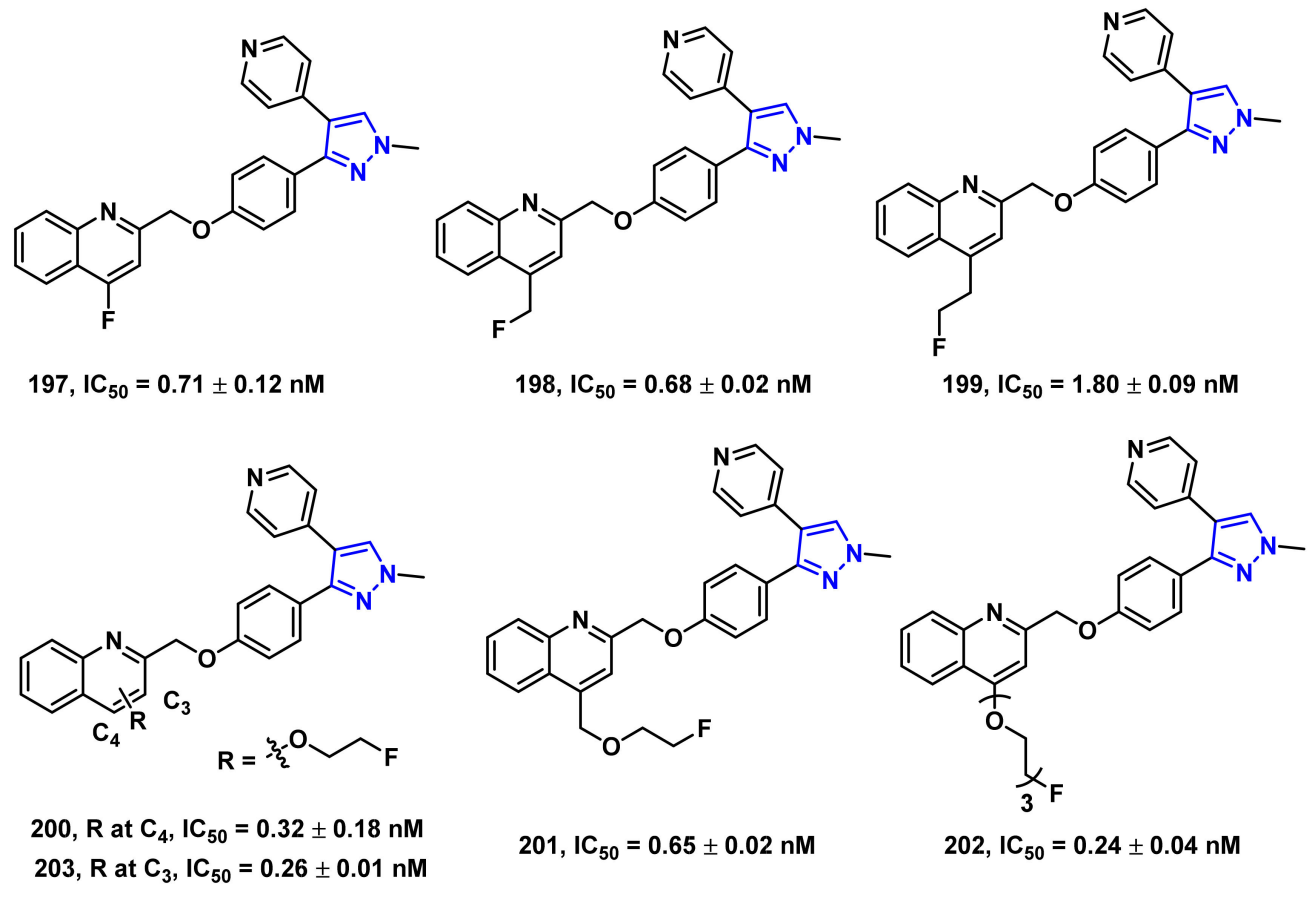
Structures of potent fluorine-containing MP-10 analogues.

In 2015, another series of potent and selective PDE10A inhibitors were reported by Tu et al. [154] (Figure 20). Fluorine-containing groups were introduced into the 2-methylquinoline moiety of MP-10. These new potent compounds (**197**–**202**) (IC_50_ range 0.24–1.80 nM) had >210-fold selectivity.

## 5. Conclusions

The wide applications of pyrazoles in pharmaceuticals have stimulated the rapid methodological development of pyrazole synthesis. During the past decade, many general and practical approaches including the involvement of transition-metal catalysts, photoredox reactions, one-pot multicomponent process, new reactants, and novel reaction type have led to fruitful advances in the fields of the synthesis and functionalization of pyrazole derivatives. This review briefly covers these updates, and highlights the potential of pyrazole scaffolds in pharmaceutical development for AD and PD treatment, two of the most serious chronic neurodegenerative diseases. The varied pyrazole compounds described herein can be regarded as candidate agents for the development of novel neurodegenerative drugs. Although in vitro and in vivo studies with promising therapeutics have shown potential to alleviate neurodegenerative symptoms, they are still far from providing a cure or effective treatment, largely due to a lack of clear understanding of disease pathologies. Therefore, studies based on rational drug design and that incorporate a deep understanding of pathological changes in neurodegenerative disease are urgently needed to explore the potential utility of pyrazoles as therapeutics, and this effort relies on multidisciplinary cooperation ranging from medicinal chemistry to pathophysiology.

## Figures and Tables

**Table 1:** Affinities of cholinesterase inhibitors.

*K_i_*^1^ or IC_50_^2^ Values for Cholinesterase Inhibitors				
Compound	R	AChE	BuChE	AChE/BuChE
**3a ^1^**	H	58.17 ± 4.84 nM	74.82 ± 18.62 nM	0.77
**3b ^1^**	Me	64.71 ± 13.04 nM	80.36 ± 22.74 nM	0.80
**3c ^1^**	Et	47.93 ± 4.92 nM	58.12 ± 9.27 nM	0.82
**3d ^1^**	Ph	44.66 ± 10.06 nM	50.36 ± 13.88 nM	0.88
**3e ^1^**	2, 5-dimethylphenyl	78.34 ± 17.83 nM	77.62 ± 18.32 nM	1.00
**3f ^1^**	3, 4-dimethylphenyl	48.16 ± 9.63 nM	65.27 ± 12.73 nM	0.73
**3g ^1^**	2-nitrophenyl	56.23 ± 11.74 nM	71.63 ± 8.93 nM	0.78
**3h ^1^**	4-bromophenyl	60.27 ± 15.67 nM	88.36 ± 20.03 nM	0.68
**164 ^1^**	--	126.13 ± 9.37 nM	145.84 ± 13.44 nM	0.86
**165 ^2^**	--	0.055 ± 0.143 µM	8.863 ± 0.22 µM	0.006
**166 ^2^**	--	0.017 ± 0.02 µM	6.331 ±0.017 µM	0.003
**167 ^2^**	--	3.148 ± 0.139 µM	1.22 ± 0.05 µM	2.58
**168 ^2^**	--	2.632 ± 0.021 µM	6.901 ± 0.01 µM	0.38
**169 ^2^**	--	0.069 ± 0.06 µM	6.3 ± 0.6 µM	0.01
**170 ^2^**	--	1937 ± 66 nM	1166 ± 88 nM	1.60

^1^ The values shown are *K_i_*, ^2^ the values shown are IC_50_

**Table 2:** Affinities of monoamine oxidases B (MAO-B) inhibitors.

	IC_50_ Value (nM)			
Compound	hMAO-A	hMAO-B	hMAO-A/hMAO-B	*K_i_* (nM)
**187**	>10,000	0.59 ± 0.09	>16,959	0.26 ± 0.04
**188**	>10,000	0.68 ± 0.04	>14,706	0.30 ± 0.02
**189**	≥10,000	0.66 ± 0.06	≥15,151	0.29 ± 0.03

**Table 3:** PDE affinities of new MP-10 analogues.

IC_50_ Value						
Compound	R	PDE10A (nM)	PDE3A (10^3^ × nM)	PDE3B (10^3^ × nM)	PDE4A (10^3^ × nM)	PDE4B (10^3^ × nM)
**195a**	3-OMe	0.40 ± 0.02	123 ± 21	82.7 ± 10	3.85 ± 0.23	3.43 ± 0.21
**195b**	4-OMe	0.28 ± 0.06	27.5 ± 2.5	3.85 ± 0.95	2.56 ± 0.11	1.79 ± 0.10
**195c**	6-OMe	1.82 ± 0.25	78.0 ± 4.0	9.75 ± 1.25	3.37 ± 0.18	2.56 ± 0.09
**196a**	3-OMe	0.24 ± 0.05	18.7 ± 3.1	16.9 ± 2.1	199 ± 16.0	31.5 ± 3.60
**196b**	4-OMe	0.36 ± 0.03	29.0 ± 4.0	4.80 ± 1.20	4.18 ± 0.33	5.06 ± 0.40
**196c**	6-OMe	1.78 ± 0.03	1.5 ± 0.5	3.00 ± 0.14	5.60 ± 0.52	7.10 ± 0.58
